# An assembly and alignment-free method of phylogeny reconstruction from next-generation sequencing data

**DOI:** 10.1186/s12864-015-1647-5

**Published:** 2015-07-14

**Authors:** Huan Fan, Anthony R. Ives, Yann Surget-Groba, Charles H. Cannon

**Affiliations:** Key Laboratory of Tropical Forest Ecology, Xishuangbanna Tropical Botanical Garden, Chinese Academy of Sciences, Mengla, Yunnan, 666303 China; University of Chinese Academy of Sciences, Beijing, 100049 China; Department of Zoology, University of Wisconsin-Madison, Madison, WI 53706 USA; Institut des Sciences de la Forêt Tempérée, Université du Québec en Outaouais, 58 rue Principale, Ripon, QC J0V 1V0 Canada; Department of Biological Sciences, Texas Tech University, Lubbock, TX 79410 USA

**Keywords:** *k-*mers, Phylogenomics, Homoplasy, Alignment-free, Assembly-free

## Abstract

**Background:**

Next-generation sequencing technologies are rapidly generating whole-genome datasets for an increasing number of organisms. However, phylogenetic reconstruction of genomic data remains difficult because *de novo* assembly for non-model genomes and multi-genome alignment are challenging.

**Results:**

To greatly simplify the analysis, we present an Assembly and Alignment-Free (AAF) method (https://sourceforge.net/projects/aaf-phylogeny) that constructs phylogenies directly from unassembled genome sequence data, bypassing both genome assembly and alignment. Using mathematical calculations, models of sequence evolution, and simulated sequencing of published genomes, we address both evolutionary and sampling issues caused by direct reconstruction, including homoplasy, sequencing errors, and incomplete sequencing coverage. From these results, we calculate the statistical properties of the pairwise distances between genomes, allowing us to optimize parameter selection and perform bootstrapping. As a test case with real data, we successfully reconstructed the phylogeny of 12 mammals using raw sequencing reads. We also applied AAF to 21 tropical tree genome datasets with low coverage to demonstrate its effectiveness on non-model organisms.

**Conclusion:**

Our AAF method opens up phylogenomics for species without an appropriate reference genome or high sequence coverage, and rapidly creates a phylogenetic framework for further analysis of genome structure and diversity among non-model organisms.

**Electronic supplementary material:**

The online version of this article (doi:10.1186/s12864-015-1647-5) contains supplementary material, which is available to authorized users.

## Background

Understanding the phylogenetic relationships among organisms is an essential aspect for many ecological, biogeographical, and evolutionary questions [1]. Currently, the simple step of generating a robust phylogeny for a group of poorly studied organisms can require substantial research investment. Most phylogenies are reconstructed from a tiny portion of the genome [2], but as next-generation sequencing technologies become faster and cheaper, the number of species for which whole genome sequence data are available has increased dramatically. Most whole-genome datasets are collected for reasons other than phylogenetic reconstruction, yet it is the first step in many comparative studies [3]. Therefore, constructing a phylogeny from genomic data would be a valuable tool even if genome datasets were not collected with this in mind. Unfortunately, most existing methods for phylogenetic reconstruction are not intended for analysis of genomic scale datasets [4]. Traditional phylogenomic techniques require genome assembly, detection of putative orthologous genes from the assembled sequences, and alignment at the DNA sequence level [5]. These analyses typically go beyond the expertise of researchers who only require a phylogeny to place comparative studies into an evolutionary framework. Approaches are needed to efficiently cope with the dramatic increase in genomic data, and to allow easy and reliable reconstruction of phylogenetic relationships among genomes [6].

Multiple-sequence alignment is a central issue in phylogenetic reconstruction, and errors in the alignment process often lead to errors in phylogenetic reconstruction [7]. When working at the genome scale, multi-sequence alignment becomes very difficult (reviewed in [8]). Alignment-free methods were initially proposed to circumvent the issues of recombination and genetic shuffling that make alignment difficult [9], and they have attracted attention due to their computational efficiency [10] and accuracy [11]. However, because they are not based on specific evolutionary models, they have been mainly used for comparing similarity between sequences [12, 13] and genomes [14].

The majority of the alignment-free methods focus on the distribution within and among study genomes of short DNA/protein fragments, known generally as *k-*mers where *k* is the length of the substring taken from the original sequences [15]. Usually distance matrices are calculated directly from the distribution of *k*-mers, and phylogenies are built from these distances [16]. These distance metrics, however, are derived without an evolutionary model and therefore do not represent the genetic distances (see [17] for a recent review). Furthermore, the *k-*mer statistics used to compare genomes are typically computed from assembled sequences [18, 19]. Unfortunately, for the majority of organisms, a reference genome from a closely related species is not available. Without a reference, *de-novo* genome assembly of short reads remains a major challenge, especially in wild, out-crossed species with high levels of heterozygosity [20]. Additionally, high coverage is generally required [21, 22], but even with high coverage *de-novo* assembly often remains error-prone [23]. The difficulty of assembly has led to methods that directly analyze unassembled read data [24, 25], and some of these methods are proposed for reconstructing phylogenies [26–29]. However, these methods are mainly designed for closely related prokaryotic genomes. Finally, although a couple of these methods [26, 29] have addressed the inherent assembly-free problems such as coverage and sequencing errors, none proposed any solution. They also do not provide methods such as bootstrapping to assess confidence in the reconstructions.

Here we present a new method that directly reconstructs a phylogeny from whole-genome short read sequence (SRS) data. By removing the need for assembly of the sequencing reads, we extend alignment-free methods to Assembly and Alignment-Free (AAF) methods. Furthermore, we develop, explain, and validate our AAF method using a combination of sequence evolution models, mathematical calculations and simulated SRS data from published genomes for 11 primates. The mathematical calculations provide the conceptual foundation for the method and predict its performance given a basic evolutionary model for sequence divergence. Simulation models of sequence evolution allow us to test the mathematical predictions. Simulations of SRS data allow us to validate the method given realistic genome complexity, different genome sizes, sequencing errors, and a range of sequence coverage. To provide a tool for researchers to assess their own AAF phylogenetic reconstructions, we also developed a two-stage bootstrap that estimates the precision of our method when applied to novel genome data. In order to demonstrate how AAF works on real sequencing data, we apply AAF to reconstruct the phylogeny for 12 mammal species from raw sequencing datasets, since the primate phylogeny is well established through both morphological and molecular data. Finally, to illustrate the ability of AAF to handle data for which it was designed – low-coverage data from poorly known species – we use AAF to reconstruct the phylogeny for 21 tropical tree species. The package is available at https://sourceforge.net/projects/aaf-phylogeny/ with detailed documentation and tutorials.

## Results and discussion

**Fig. 1:**
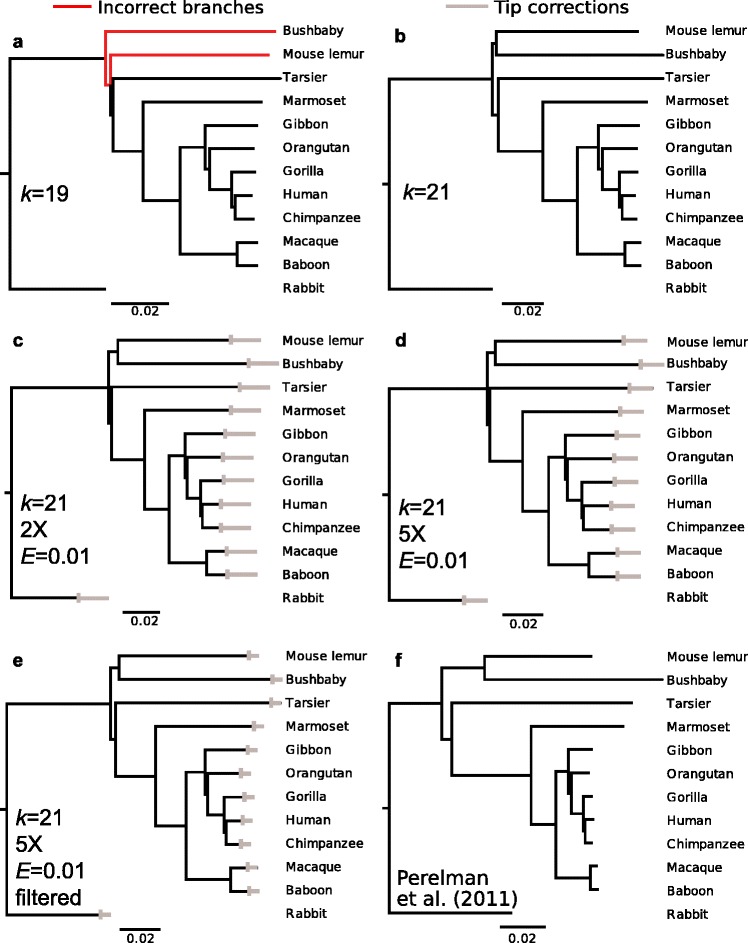
Primate phylogeny reconstructed by AAF using assembled genomes and simulated short reads. **a** Phylogeny reconstructed from assembled genomes with *k* = 19. Incorrect branches are shown in red. **b** Phylogeny reconstructed from assembled genomes with *k* = 21. For the assembled genomes, correct phylogenies were also given for *k* = 23, 25 and 31 (Additional file 1: Figure S1). **c** Phylogeny reconstructed from 70-bp reads were simulated from assembled genomes with 1 % sequencing errors and 2X coverage. Grey bars at the tips give the tip corrections (Eq. 10) for incomplete coverage and sequencing error that are trimmed from each tip.**d** Like (**c**) but with 5X coverage. **e** Like (**d**) but with filtering to remove *k*-mers that appear only once. **f** A recently published phylogeny of primates [31]

The AAF approach first calculates pairwise genetic distances between each sample using the number of evolutionary changes between their genomes, which are represented by the number of *k-*mers that differ between genomes. The phylogenetic relationships among the genomes are then reconstructed from the pairwise distance matrix. Using simulated SRS read data (with sequencing error and incomplete coverage) from published and fully assembled genomes, our AAF method obtained the same phylogeny for 11 primate species (plus one outgroup, Fig. 1) as those previously published using traditional methods [30, 31], even though AAF did not use any information about assembly or alignment. Furthermore, the AAF method was very efficient, requiring only a few days on a standard work station (Table 1). Below, we provide complete theoretical and computational support for our method.

**Table 1:** AAF performance metrics

Coverage	Filtered	Memory (MB)	Generating *k*-mer table (CPU hours, using one core)	Calculating distance matrix (CPU hours, using one core)
5X	no	68,082	117	103
5X	yes	68,086	85	56
2X	no	39,780	66	61
2X	yes	39,048	39	22
1X	no	34,316	50	8

Performance metrics for the AAF reconstruction of the 11-primate phylogeny from simulated SRS data using one thread only. Phylogenetic reconstruction requires generating the *k*-mer table and calculating the number of shared *k*-mers between species to compute the distance matrix. Note that the memory usage is for the first step; the second step required no more than 1G of memory

### Evolutionary model

Our measure of the phylogenetic distance (denoted *d*) between two species, A and B, is based on the estimate of the rate parameter from a Poisson process for a mutation occurring at a single nucleotide under the evolutionary model that the mutation rate is the same for all nucleotides across the genomes. We include not only mutations caused by nucleotide substitutions, but also insertions and deletions (indels). If *k-*mers are random nucleotide sequences of length *k*, and if mutations occur randomly and independently among nucleotides, the probability that no mutation will occur within a given *k-*mer between species A and B is exp(−*kd*). Mutations will decrease the number of shared *k-*mers, *n*_*s*_, between species relative to the total number of *k-*mers, *n*_*t*_. In the hypothetical case in which only substitutions occurred and all *k-*mers were unique, then all the species will have the same total number of *k-*mers, *n*_*t*_, and the maximum likelihood estimate of exp(−*kd*) is *n*_*s*_/*n*_*t*_. In real situations, indels introduce the complication of changing *n*_*t*_ which can be addressed as follows. A single insertion of length *l* will cause the loss of at most (*k* – 1) *k-*mers and a gain of at most (*l* + *k* – 1) *k-*mers, while a single deletion of length *l* will cause the loss of at most (*l* + *k* – 1) *k-*mers and a gain of at most (*k* – 1) *k-*mers. For computing distances, this means that deletions will cause a greater reduction in the number of shared *k-*mers than either a substitution or an insertion of the same length. To account for this asymmetry, when estimating the distance between two taxa, the smaller of the two values of *n*_*t*_ calculated separately for each of the taxa is used. This leads to the estimate of *d* (denoted *D*) of

1 $$ D=\frac{-1}{k} \log \frac{n_s}{n_t} $$

This formula can be modified to correct for back substitutions (reversal to a nucleotide’s original state after two or more mutations), although this effect is small (see Methods: Estimating *d*).

Although application of Equation 1 for AAF is in principle straightforward, several important issues must be addressed. These divide naturally into alignment-free and assembly-free issues. Below, we first address alignment-free issues that involve extracting as much information as possible about the true evolutionary variation between species. We then address assembly-free issues that involve reducing sampling variation caused by incomplete coverage and sequencing errors.

### Alignment-free: *k*-mer sensitivity and homoplasy

Lack of alignment makes it more difficult to extract all of the possible information about evolutionary distances between species. If genomes from two species were perfectly aligned, it would be possible to identify all substitutions and indels. In AAF, however, only differences in the presence/absence of *k*-mers are used. If a *k*-mer covers, for example, multiple substitutions, it will count equally as one carrying only a single substitution. Consequently, shorter *k*-mers are more likely to have greater sensitivity to single evolutionary events. On the other hand, identical *k*-mers could be derived from physically, functionally, or evolutionarily different regions of the genome and are therefore not homologous (*k*-mer homoplasy). Longer *k*-mers are less likely to suffer from *k*-mer homoplasy. Therefore, a trade-off exists for *k*-mer length between the problem of sensitivity (which requires smaller *k*) and *k*-mer homoplasy (which requires larger *k*).

To isolate alignment-free issues, in this section we compute *k*-mers under the assumption that we have completely assembled genomes without error. Therefore, only the evolutionary differences between genomes will be captured, without any differences caused by sampling error or sequencing effort.

#### k-mer homoplasy

*k-*mer homoplasy is generally considered as “noise” in phylogenetic reconstruction [16] and is a particular problem when *k-*mer length is short and genome size is large. For example, if *k* = 15, the total number of possible *k-*mers accounting for complementarity is 4^15^/2 (~5x10^8^), so if genomes are close to this length, identical *k-*mers will appear in different species simply due to the limited number of total possible *k-*mers.

*k-*mer homoplasy may incorrectly inflate the proportion of shared *k-*mers because (i) multiple copies of the same *k*-mer at different locations in species A must all experience mutations before this *k-*mer is no longer shared with species B, and (ii) a *k-*mer that does undergo a mutation may turn into a *k-*mer that already exists elsewhere in the genomes of species A or B (see Methods: *k*-mer homoplasy). *k*-mer homoplasy depends on the frequency distribution of different *k-*mers across the genome of species A and B, which varies with *k*; for the species with the shortest genome, we denote this distribution *Q*_*k*_. To account for *k*-mer homoplasy, we derived a mathematical formula for the proportion of shared *k-*mers between species, *p*_*h*_, which is a prediction of the ratio *n*_*s*_/*n*_*t*_ based on *Q*_*k*_ (Methods: *k-*mer homoplasy). In this formula, *Q*_*k*_ can be calculated empirically from real genomes (Eq. 4) or estimated theoretically under the assumption that the ancestral genome for species A and B is a random sequence (Eq. 5).

**Fig. 2:**
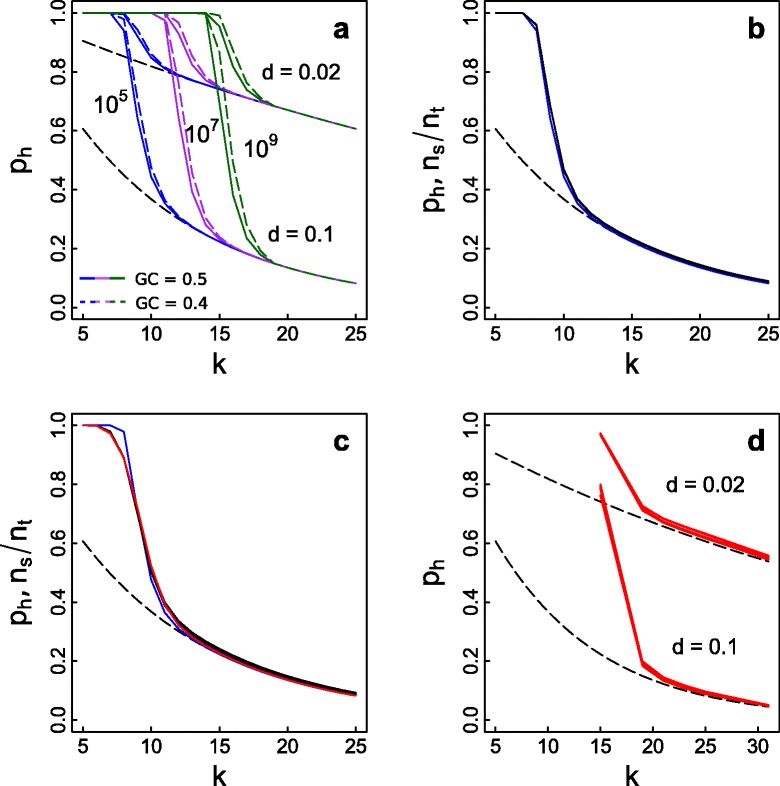
Effect of *k-mer* length on *k*-mer homoplasy. **a** Mathematical predictions of the proportion of shared *k*-mers, *p*_*h*_, as a function of *k* for genomes of sizes *g* = 10^5^ (blue), 10^7^ (purple), and 10^9^ (green) when the true genetic distance between two species is *d* = 0.02 or *d* = 0.1, and the GC content is 0.5 (solid lines) and 0.4 (dashed lines). The dashed black line gives the hypothetical case if there were no *k*-mer homoplasy. Calculations were performed using the assumption that genomes are random sequences (Eq. 5). **b** Simulations of the effect of *k*-mer homoplasy on *n*_*s*_/*n*_*t*_ and comparison with its theoretical prediction *p*_*h*_. Three simulations were performed starting with a random sequence of 10^5^ bp assuming that the true genetic distance between taxa is *d* = 0.1. The black lines give *n*_*s*_/*n*_*t*_ from sequence simulations and the blue lines give the theoretical predictions, *p*_*h*_, under the assumption that the ancestral genome is random with GC content 0.5 (Eq. 5). **c** Like (**b**) but with the ancestral sequences given at three random starting positions from a published 1.9 Mbp sequence of the rabbit genome [30]. The red line gives the theoretical predictions (Eq. 4) calculated using the observed frequency distribution of *k*-mers, *Q*_*k*_, in one of the simulated species. **d** Theoretical predictions (Eq. 4) of the proportion of shared *k*-mers, *p*_*h*_, calculated from the observed frequency distribution of *k*-mers, *Q*_*k*_, for the 11 primate genomes ranging in size from 2.7 to 3.5 Gbp assuming the true distance between taxa is *d* = 0.02 or 0.1

We first investigated the general issue of *k-*mer homoplasy under the assumption that the ancestral genome is a random sequence (Fig. 2a). For large genomes and small *k*, *k-*mer homoplasy led to *p*_*h*_ = 1 because all possible *k-*mers occur in both species. This problem is exacerbated if GC content is biased, which will inflate the average similarity in genomic *k-*mer composition. Fortunately, because the possible number of *k*-mers increases exponentially, small increases in *k* quickly overcome this limitation; for example, when *k* = 18 over 30 billion *k*-mers are possible, which is considerably larger than the majority of genome sizes, and when *k* = 21, over 2 trillion possible *k*-mers exist. Therefore, above a threshold *k* (which differs by genome size and sequence complexity), the effects of *k-*mer homoplasy are greatly reduced. As would be expected, above this *k* the theoretical and observed values of *p*_*h*_ are the same (Fig. 2a dashed black line). Furthermore, the *k* at which *k-*mer homoplasy vanishes depends only weakly on the genetic distance *d* between species (Fig. 2a, *d* = 0.02 vs. *d* = 0.1). Therefore, a sufficiently large *k* will overcome homoplasy, regardless of the evolutionary distance between species.

The mathematical formula for *p*_*h*_ accurately predicted the results from simulated sequence evolution starting from either a random ancestral sequence of 100 kbp (Fig. 2b) or real (non-random) sequences (Fig. 2c). Comparing sequence evolution simulated from random and real ancestral sequences, *k* must be larger to reduce *k-*mer homoplasy with the real ancestral sequence (Fig. 2c); this is because the lower complexity in the real ancestral sequence increases the probability that a *k-*mer appears at multiple locations in the genome by chance. For random sequences of length 10^9^ bp, *k-*mer homoplasy is negligible for *k* ≥ 19 (Fig. 2a), whereas for *Q*_*k*_ obtained from the primate genomes, *k-*mer homoplasy only becomes negligible for *k* ≥ 21 (Fig. 2d).

#### Balancing sensitivity and homoplasy

While *k-mer* homoplasy becomes negligible when *k* is sufficiently large, continuing to increase *k* will also increase the probability that a single *k-*mer contains more than one evolutionary event, and this will reduce sensitivity and underestimate the proportion of shared *k-*mers.

**Fig. 3:**
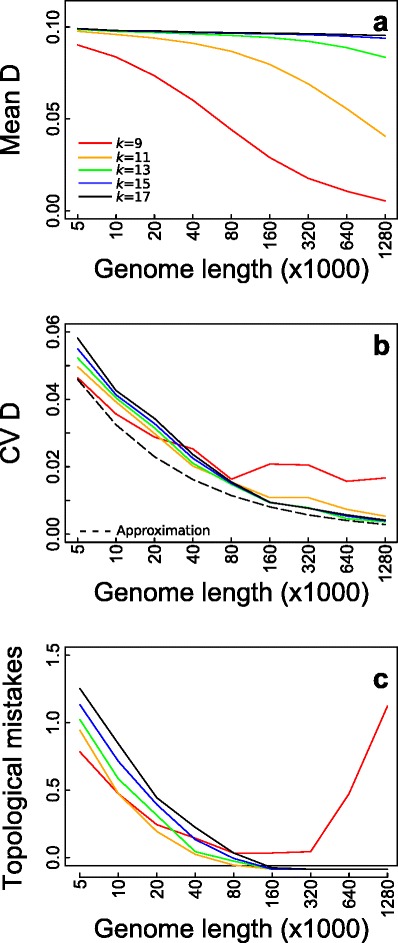
Statistical properties of bias and precision of the estimate *D* caused by alignment-free issues only*.* **a** Mean and (**b**) coefficient of variation (CV) of *D* between two species as a function of genome size. *k-*mer lengths are *k* = 9 (red), 11 (orange), 13 (green), 15 (blue) and 17 (black). The true distance between species is *d* = 0.1. In (**b**) the dashed line is the approximate CV of *D* calculated assuming that all mutations were identified. **c** Average number of topological mistakes generated by AAF from simulated sequences on the phylogeny depicted in Fig. 1**b**, with different ancestral genome lengths and different *k*. One hundred simulations were performed for each length using ancestral genomes taken from random starting positions on a 1.9 Mbp sequence of the rabbit genome from Prasad et al. (2008)

The balance between *k-*mer homoplasy and sensitivity can be understood in terms of the statistical properties of bias and precision in the estimate *D* (Fig. 3). Bias refers to the under- or overestimation of distances, whereas precision refers to the variability in the estimates. We investigated both the bias and precision by simulating sequence evolution (Methods: Simulation of sequence evolution); for these simulations, we used relatively short ancestral sequences (compared to the primate genomes) for which smaller values of *k* will be enough to overcome *k*-mer homoplasy. For short *k-*mers (*k* = 9, 11), increasing genome size led to consistent underestimates of *D* (Fig. 3a) due to the increased numbers of shared *k-*mers from *k-*mer homoplasy (Fig. 2). At small genome sizes, however, shorter *k-*mers led to greater precision in the estimate *D* measured by the coefficient of variation, CV (Fig. 3b). This greater precision for shorter *k-*mers occurred because shorter *k-*mers have greater sensitivity to identify individual mutations. At large genome sizes, however, the precision decreased for shorter *k-*mers due to *k-*mer homoplasy. These results predict that shorter *k-*mers will give better phylogenetic reconstructions up to the point that *k-*mer homoplasy leads to strong bias and imprecision in the estimate *D*. Therefore, the optimal *k* for phylogenetic reconstruction is the *k* which is just large enough to greatly reduce *k-*mer homoplasy for a given genome size (Fig. 2).

To test this conclusion, we simulated 12 sequences based on their phylogenetic relationships starting with ancestral sequences ranging from 5 kb to 1280 kbp (100 phylogenies simulated for each starting length) given the “true” phylogenetic relationships among simulated species are given by Fig. 1b. As expected, for short genomes for which *k-*mer homoplasy was negligible, shorter *k-*mers led to fewer topological mistakes in phylogeny reconstruction (Fig. 3c). However, as genome length increased, phylogenies reconstructed using *k* = 9 possessed an increasing number of mistakes. For longer *k-*mers (11 ≤ *k* ≤ 17), AAF invariably gave the correct topology when genome size was ≥160 kbp. Furthermore, the adequate performance with *k* = 11 despite the expected underestimate of *D* (Fig. 3a) suggests that reconstructing tree topology is robust to moderate amounts of bias.

#### Phylogeny reconstruction from assembled genomes

Using 11 assembled primate genomes with rabbit as an outgroup, AAF with *k* = 21 generated a phylogeny with the same topology as those described in recent publications [30, 31]. With *k* < 21, a few topology errors were observed, especially for deep nodes; these errors were anticipated as a result of *k*-mer homoplasy (Fig. 2c). For *k* > 21, tree topology (Additional file 1: Figure S1) and branch lengths (Additional file 2: Table S1) were remarkably stable. This matches our prediction from Fig. 2c that values of *k* ≥ 21 should be sufficient to minimize *k*-mer homoplasy. Therefore, we take 21 as the optimal *k* that balances homoplasy and sensitivity for this dataset, and we take the tree constructed from assembled genomes with optimal *k* as our optimal AAF tree (Fig. 1b). Quantitative comparison with the phylogeny from Perelman et al. (2011) showed high similarity to our optimal phylogeny with respect to branch lengths; there was a high correlation between their patristic distances (r = 0.9717) and a low Branch Score Distance (BSD = 0.0518;[32]). AAF is also very efficient, requiring only a few days on a standard workstation (Table 1).

### Assembly-free: incomplete coverage, sequencing error, and filtering

While the lack of alignment introduces possible errors in the inference of the actual evolutionary relationships among species, the lack of assembly primarily introduces sampling error caused by low genome coverage and sequencing errors [26, 29]. The actual number of *k-*mers will be under-represented given low sequencing coverage, whereas sequencing error will cause both the loss of true *k-*mers and the gain of false *k-*mers.

One simple solution to remove false *k-*mers caused by sequencing error is to filter out all low-frequency *k*-mers; here, we filtered by removing *k*-mers that occur as singletons (those which occur as single copies) in a genome. If sequencing errors are random, with no molecular or experimental bias, then the probability of observing the identical sequencing error at the same position is low. Therefore, given observed error rates for most next-generation sequencing platforms, *k-*mers observed more than one time in the SRS data for a genome are unlikely to be errors. However, as sequencing coverage decreases, a larger fraction of real *k*-mers will be singletons in the dataset, and therefore filtering will remove real *k*-mers. As a consequence, although filtering will be beneficial at high coverage, at low coverage filtering will be detrimental. We investigated incomplete coverage, sequencing error, and *k-*mer filtering using mathematical calculations, simulations, and application to simulated SRS data to determine the severity of potential problems caused by sampling errors and to derive recommendations accordingly.

#### *Total and shared* k*-mers with missing and false* k*-mers*

**Fig. 4:**
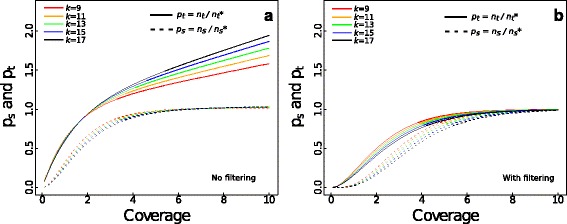
The theoretical ratios of observed to true total and shared *k-*mers. **a** Ratio of observed to true total *k*-mers, *p*_*t*_ *= n*_*t*_/*n*_t_* (solid lines) and ratio of observed to true shared *k*-mers, *p*_*s*_ = *n*_*s*_/*n*_s_* (dashed lines), when *k-*mers are not filtered (Methods: Combined effects of coverage and sequencing error). *k-*mer lengths are *k* = 9 (red), 11 (orange), 13 (green), 15 (blue) and 17 (black). The simulation was performed with a sequencing error rate of 1 % and a read length of 76 bp, and the true distance between species is *d* = 0.1. **b** Like (**a**) but with filtering

When there is incomplete coverage and sequencing errors, the true total and shared numbers of *k-*mers, denoted *n*_t_* and *n*_s_*, differ from the observed total and shared *k-*mers, *n*_*t*_ and *n*_*s*_. We derived mathematical formulas (Methods: Combined effects of coverage and sequencing error) to predict the ratios *p*_*t*_ = *n*_*t*_/*n*_t_* and *p*_*s*_ = *n*_*s*_/*n*_s_* given information about coverage, sequencing errors, and *k-*mer filtering (Fig. 4). False *k-*mers caused by sequencing errors inflate *p*_*t*_ starting from 2X coverage (Fig. 4a, solid lines), and at least 5X coverage is required to capture most of the shared *k*-mers (dashed lines). When filtering out singleton *k*-mers, *p*_*t*_ at higher coverage correctly equals one; however, at lower coverage *p*_*s*_ is reduced by 20-30 % (Fig. 4b) due to the loss of true singletons. Thus, the advantage of filtering is that it reduces the number of false total *k-*mers, at the cost of losing true shared *k-*mers.

#### To filter or not to filter?

**Fig. 5:**
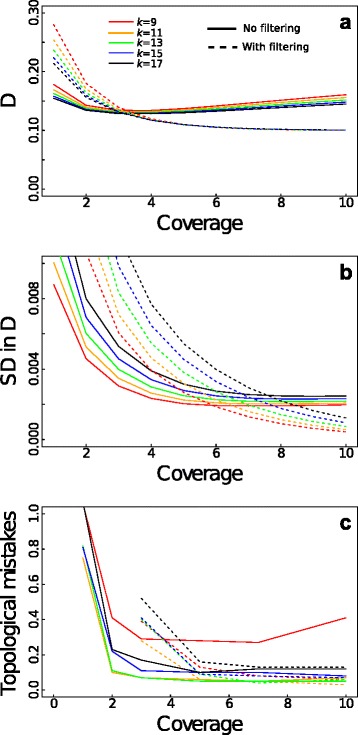
Statistical properties of the estimate *D* with combined effect from assembly and alignment-free issues. **a** Mean and (**b**) standard deviation in the estimate *D* from simulations with incomplete coverage and 1 % sequencing error. The true distance between species is *d* = 0.1, and *k-*mer lengths are *k* = 9 (red), 11 (orange), 13 (green), 15 (blue) and 17 (black), with solid and dashed lines corresponding to no filtering and filtering, respectively. **c** Average number of topological mistakes generated by AAF from simulated sequences on the phylogeny depicted in Fig. 1**b**. One hundred simulations were performed using 80 kbp of a 1.9 Mbp sequence of the rabbit genome from Prasad et al. (2008) with random starting positions. For each sequence simulation, reads of length 76 were simulated and each read dataset was analyzed using different *k-*mer lengths

Given the trade-off between filtering and not filtering, we investigated how much coverage is required before filtering should be used. Whether or not to filter *k-*mers can again be decided by computing the bias and precision of the estimate *D* (Eqs. 8 and 9). Without filtering, the number of false *k-*mers increases, and the true distance between species is overestimated (Fig. 5a, solid lines). Filtering out singletons can correct for this sequencing error effect with sufficient coverage (5-8X according to genome size, dashed lines).

Because reconstructing the correct topology appears robust to bias in the estimate *D* (Fig. 3a vs 3c), precision is likely more important, and filtering should be done when it can lower the variance in the estimate *D*. This crossover point generally occurs between 5X and 8X, depending on *k* (Fig. 5b, dashed lines for filtering and solid lines for no filtering).

To confirm these conclusions, we simulated SRS data with sequencing error and different coverage using ancestral genomes of length 80 kbp (Fig. 5c). As predicted by the estimates of the precision (Fig. 5b), filtering led to fewer mistakes in the topology of the phylogeny when coverage exceeded a threshold between 5X and 8X. The poor performance with *k* = 9 (Fig. 5c, red line) was due to *k-*mer homoplasy as found previously (Fig. 2b). With the genome size of 80 kbp, *k* = 11 and 13 (Fig. 5c, orange and green lines) performed slightly better than other values of *k* in the simulations of assembled genomes (Fig. 3c) and here had the additional advantage of being less sensitive to sequencing error (Fig. 5a, b).

### Tip correction

Incomplete coverage and sequencing error both inflate estimated distances between species and lead to longer tips on the tree. We derived a mathematical correction for this effect (Eq. 10) that does not depend on the true distance between species, but does depend on the read length and sequencing error and coverage. When the read length, sequencing error, and coverage are similar for all taxa, then this correction is similar for all species, so that the correction only affects the tips rather than internal nodes of the tree. This correction is particularly helpful for lower coverage datasets when filtering is not an option. Note that the decision whether to filter depends on precision (Fig. 5); therefore, although the tip correction adjusts for bias, it does not affect the decision whether to filter. The case of large differences in read length, sequencing error, and coverage among taxa is discussion in the Methods: Tip correction.

### Bootstrapping

To determine the uncertainty in tree topology, we devised a two-stage nonparametric bootstrap that accounts for both sampling variation (caused by incomplete coverage and sequencing error) and evolutionary variation (caused by the true history of sequence divergence and the ability of AAF to reconstruct it). The first stage of the bootstrap follows standard procedures: resample the original reads with replacement, construct a *k-*mer presence/absence table, compute distances *D*, and construct the phylogeny. The variance in topology among 100 replicates is then used to assess the precision of reconstruction associated with sampling variation. To additionally incorporate evolutionary variation, the second stage involves, for each of the 100 *k-*mer presence/absence tables, resampling with replacement 1/*k* of the total *k-*mers. Only 1 in *k* of the *k-*mers is selected to account for the non-independence among *k-*mers caused by their overlap. Simulations showed that choosing 1/*k* of the *k-*mers gives the correct variance in *D* in the bootstrap (Methods: Bootstrapping).

**Fig. 6:**
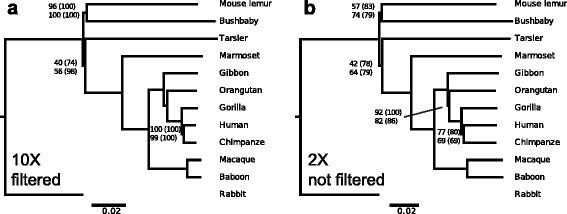
Two-stage nonparametric and parametric bootstraps. **a** Examples of two-stage nonparametric and parametric bootstraps from simulated data with high coverage (10X) and filtering (*k* = 13). The node values give the number of bootstrap trees supporting each node, with the values in parentheses giving bootstraps accounting for only sampling variability (first stage); for each node, the top numbers give the results from the nonparametric bootstrap and the bottom numbers give the results of the parametric bootstrap. Nodes without values all have 100 % support. **b** like (**a**) but with low coverage (2X) and without filtering. The ancestral genomes (different between panels) were 80 kbp sequences taken from random starting positions on a 1.9 Mbp sequence of the rabbit genome from Prasad et al. (2008)

This nonparametric bootstrap is not practical for large genomes due to the computational requirements for repeating the phylogenetic reconstruction a hundred or more times. Therefore, we also developed a two-stage parametric bootstrap that uses mathematical equations to estimate the variances in distances between species caused by sampling and evolutionary variation (Methods: Bootstrapping). This approach gives similar results to the nonparametric bootstrap in both simulations with high coverage and filtering (Fig. 6a), and low coverage without filtering (Fig. 6b), but its computational requirements are independent of genome size.

### Application on simulated and raw sequencing data

As an initial test of AAF, we simulated reads from 11 fully assembled primate genomes with rabbits as an outgroup (Methods: Read simulations). AAF successfully reconstructed phylogenies with the same topology and only slightly different branch lengths from simulated SRS data (Fig. 1c-e, Table 2). The closest match to the phylogeny produced from the assembled primate genomes with *k* = 21 (Fig. 1b) was the filtered 5X coverage (Fig. 1e, BSD = 0.016), followed by the non-filtered 2X coverage (Fig. 1c, BSD = 0.063) and then non-filtered 5X coverage (Fig. 1d, BSD = 0.10). In fact, even for 0.5X coverage and no filtering, AAF recovered the correct topology (Additional file 1: Figure S1e), although we do not recommend application of AAF to datasets with such low coverage. Finally, with the tip correction, the trees are more similar to those constructed from the fully assembled genomes (Table 2).

**Table 2:** Branch Score Distance (BSD) between trees generated from simulated reads and the optimal AAF tree

Tree	No tip correction	With tip correction
AAF 2X (Fig. 1c)	0.062	0.022
AAF 2X filtered	0.063	0.043
AAF 5X (Fig. 1d)	0.104	0.028
AAF 5X filtered (Fig. 1e)	0.016	0.011

The optimal AAF tree that is generated from assembled data using *k* = 21 for the 11 primate dataset (Fig. 1B) is compared to trees generated from simulated SRS data with different coverage, with or without filtering, and before and after tip correction

Although we have included sequencing error and random sequences in our read simulations, there are non-random biases brought by different sequencing technologies. In order to test the performance of AAF on real sequencing data, we downloaded next-generation sequencing data for 7 primates that are available in NCBI Short Reads Archive. We did not find data for rabbit in SRA, and therefore we used cat as the outgroup. We added five species to expand the study to Euarchontoglires, the super-order to which primates belong. This dataset includes sequences from different sequencing platforms with different library construction strategies and read lengths (Additional file 3: Table S2). AAF successfully reconstructed a phylogeny from the 12 mammals dataset (Additional file 4: Figure S2) that agrees with recent studies [33, 34]. This illustrates how AAF can be used to construct phylogenies from publicly available, heterogeneous genome data.

**Fig. 7:**
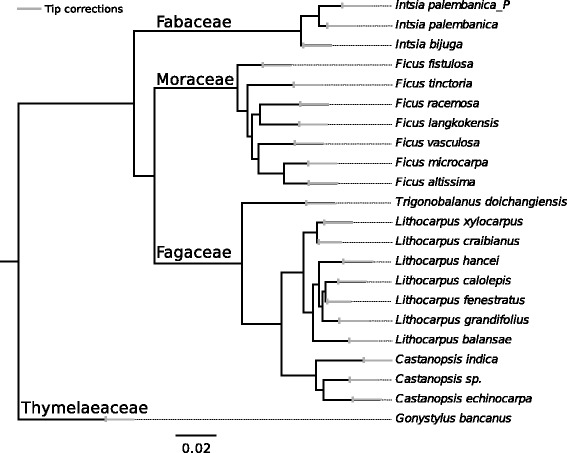
AAF phylogeny of 21 tropical trees constructed from raw reads with *k* = 27. Grey bars at the tip branches give the tip corrections (Eq. 10) for incomplete coverage and sequencing error that are trimmed from each tip. *Intsia palembanica*_P is a pooled sample containing 10 individuals. Both the construction of the tree and tip correction are presented in Additional file 6: Tutorial of the analysis of the 21 tropical trees dataset

AAF is designed mainly for constructing phylogenies for non-model organisms. Therefore, we showcase AAF on raw SRS datasets that might be typically encountered by evolutionary biologists and ecologists, a dataset of 21 tropical trees from four orders (Additional file 5: Table S3). Analyzing this 21 tropical trees dataset was the initial stimulus for developing AAF, and the dataset contains the many complications that will arise in studies of non-model organisms: low and uneven coverage, different read lengths, and no available reference genome close enough to aid traditional assembly and alignment. The AAF tree (Fig. 7) matched the established phylogeny at the interfamily level according to the current Angiosperm Phylogeny Group classification, APGIII [35], and the intergenera level according to recent studies [36, 37]. However, at the intragenus level, there is no published phylogeny for one of the major genera, *Lithocarpus;* the existing phylogenies for the other major genus, *Ficus,* have no consensus and poor bootstrap support [38, 39]. Therefore, we cannot compare our results diagnostically to established results in the literature. A tutorial giving a full demonstration from parameter selection to phylogeny reconstruction, tip correction and bootstrapping of this dataset is provided in the Additional file 6: Tutorial of the analysis of the 21 tropical trees dataset.

### Comparison with other alignment and assembly-free methods

Although there are other alignment and assembly-free methods for constructing phylogenies, these methods are designed for prokaryote genomes [27, 28, 40]. A major advantage of AAF is its ability to process large genomes within days. The only methods that demonstrated attempts on mammalian-size genomes are provided by Song et al. [26] and Yi and Jin [29].

Song et al. [26] extended a variant of the classic alignment-free statistic, D_2_, to perform assembly-free phylogenetic reconstructions. The authors demonstrated its application on 1X simulated genome sequences of five mammals and raw sequences of 13 tropical trees (included within the 21 species we present in our Tutorial), but the results were not convincing. For mammals, none of the resulting clustering was consistent with the known phylogenetic relationships of the five species. For the tropical trees, species from the same genus (*Lithocarpus*) were not grouped together, and the relationship between genera within Fagaceae was not consistent with previous studies [41]. Song et al. [26] did not provide a package which we could use for direct comparisons with AAF, although our successful application to 12 mammals and 21 tropical tree species contrasts with their results.

Yi and Jin [29] developed *co-phylog* that uses an micro-alignment method for identifying SNPs (http://humpopgenfudan.cn/resources/softwares/CO-phylog.tar.gz.). This method is designed primarily for closely related species, and the applications Yi and Jin present consist mainly of bacteria. The authors applied *co-phylog* to 5 mammal species in one of their supplemental figures. However, when we tried to repeat this analysis, the intermediate file (.co file) for a single run of one species (Bushbaby, SRR953063) was 129G. Ideally the intermediate file could be limited to around 24G for mammal species (genome size times 8byte), only if given perfectly assembled genomes without repetitive segments. Since each species has dozens to hundreds of runs of data, we were not able to replicate their results and compare their method to AAF based on real large genome data. This type of analysis will require more hard drive space than most potential users will have available, and this limits the practical use of the method for large genomes.

For a direct comparison between AAF and *co-phylog* for smaller datasets, we simulated 100 data sets and assessed the performance of the two methods by the number of topological mistakes in the phylogeny. This simulation study is similar to that in Fig. 3c (see Methods: Simulation of sequence evolution). To select the length of the flanking regions (CK) to use in *co-phylog* (since no guidance is provided with the method), we used our calculations for optimal *k* to avoid homoplasy, which should pose the same problems for *co-phylog* as for AAF; thus, in *co-phylog* we set the combined length of the flanking regions plus the focal nucleotide (i.e., 2*CK* + 1) equal to *k* in AAF. AAF uniformly outperformed *co-phylog* (Additional file 7: Figure S3). Furthermore, the performance of *co-phylog* depended on *CK* in a manner predicted for the selection of *k* in AAF: for short initial sequences, smaller values of *CK* performed best because they are more able to identify SNPs that are located close together on the genome. However, for longer sequences performance was degraded by homoplasy for small *CK*. The best value of *CK* for genome sizes >160 KB was *CK* = 6, which is comparable to *k* = 13 in AAF.

### Advantages of the AAF method

The AAF method greatly simplifies phylogenetic reconstruction from genomic scale data. Compared to traditional phylogenetic methods, the AAF method uses the evolutionary signal from the whole genome, including coding and non-coding regions, and small-scale structural differences, like indels, not just nucleotide substitutions. In contrast to most current alignment-free methods, the distance matrix calculated by AAF matches with the genetic distance. In contrast to the few available assembly-free methods, AAF can analyze large genomes and huge datasets while accounting for problems posed by lack of assembly, specifically sequencing read errors and incomplete coverage. Our strategy of treating *k*-mers as present/absent instead of using their absolute or relative frequencies will be advantageous for the direct analysis of raw SRS data, particularly when the coverage varies within and among genomes. Using presence/absence of *k*-mers also makes AAF more robust to repetitive sequences with high abundance. We demonstrated that the method has statistically well-defined properties that allow optimization and adaptation of the method. These properties also provide predictive insight into the strengths and weaknesses of AAF given a range of evolutionary and sampling conditions.

Other main advantages of the AAF method include:

(i). Low coverage requirements. Even with the increasing throughput of next-generation sequencing technology, it is still expensive to sequence many genomes at the high coverage needed for successful assembly. The AAF method is able to recover an accurate phylogeny with low coverage (Fig. 5c). This is largely due to the abundance of information brought by whole genomes. Even for species with large genomes like primates, it is possible to obtain 2X coverage for five individuals from a single Illumina HiSeq lane. Therefore, AAF can generate a robust phylogeny quickly and inexpensively for any group of species of interest.
(ii). Low computational demands. Traditional phylogenomic studies rely on three main steps (without considering data acquisition) that are computationally demanding: assembly, orthologous gene identification and alignment, and phylogenetic reconstruction based on the aligned sequences. Traditional phylogenetic methods like maximum-likelihood and Bayesian methods rely on complex evolutionary models that require large amounts of computing time, even when using large computing clusters. The AAF method decreases the total analysis time drastically. Compared to 40–50 h required for human genome assembly using a supercomputer [42], which is just the first step of traditional analysis for one species, AAF takes less than two days to complete the phylogenetic reconstruction of a dozen primate genomes on a standard workstation with 25G of RAM and 12 threads.

### Limitations

The advantages of the AAF method come with some costs:

(i). Loss of *k*-mer sensitivity. AAF does not use all of the evolutionary information that would be available if genomes were accurately assembled and aligned. The minimum length of *k*-mers is set by the need to overcome *k*-mer homoplasy; for the primate dataset in this study, the minimum length was *k* = 21. Therefore, if there are multiple mutations (substitutions or indels) within a 21-nucleotide sequence, they will be covered by the same *k*-mer. Nonetheless, the vast amounts of data available from entire genomes will largely overcome this problem.
(ii). Deep nodes. The accuracy of most alignment-free methods suffers when applied to sequences or genomes separated by large genetic distances [27, 29]. Deep phylogenetic nodes imply a higher density of mutations. This will cause the loss of sensitivity of AAF to differences between genomes, and will also exacerbate the effects of homoplasy, which in turn will decrease the estimate *D* between distantly related species (Additional file 8: Figure S4). In our simulations and application to the primate genomes, AAF performed well despite a divergence time for primates estimated at 87 Mya [31] and estimated genetic distance (probability of mutation) from basal node to tips of roughly 0.1. It also worked well when expanding the analysis to Euarchontoglires (Additional file 4: Figure S2b). However, AAF failed to identify some of the deep nodes when including more mammals across the Placentalia clade (Additional file 4: Figure S2c) with a divergence time of >100 Mya [33]. In our dataset of 21 tropical trees, the AAF relationships between families were consistent with the APG III System. The divergence time for this group is about 94 Mya, and the average genetic distance from basal node to tips is roughly 0.1 as well. These empirical examples suggest that AAF can successfully reconstruct phylogenies with divergence times of <100 Mya and genetic distances from base to tips of <0.1, and we do not recommend AAF for deeper nodes.
(iii). Location of mutations. AAF does not directly give information about where genetic differences between genomes occur. Specifically, the calculation of the phylogeny from the distance matrix does not estimate ancestral states that might be used for mapping specific mutations onto the phylogenetic tree. Thus, AAF does not identify genes or regions of the genome that are conserved or discordant. Nonetheless, once a phylogeny is constructed, analyzing distribution patterns of genes and genome regions across the phylogeny can be performed on *k-*mers directly [25, 43]. Integration with AAF, however, will require additional development.

### Guidelines for parameter selection

AAF requires two choices from the user: (i) what *k-*mer length to use, and (ii) whether to filter out singleton *k-*mers. The choice of *k* depends on the possibility of *k-*mer homoplasy; *k* must be long enough to guard against it. This choice can be made for real genomes by plotting *p*_*h*_ and selecting *k* where the estimated value of *p*_*h*_ matches the theoretical value assuming no *k-*mer homoplasy, using empirically calculated *Q*_*k*_ (Fig. 2d, Additional file 9: Figure S5). Larger values of *k* beyond this will generally decrease the performance of AAF due to both the loss of sensitivity (from covering multiple mutations under the same *k-*mer) and the increased likelihood of *k-*mer loss through sequencing error if *k-*mers are not filtered (Fig. 4). We have included the R code for plotting Fig. 2d to provide a starting point for the selection of *k* (see more details in the tutorial). To confirm this selection, users can repeat the analyses while increasing *k* by 2 until successive values of *k* give the same phylogeny.

Filtering is a good guard against sequencing error and inflated tip lengths in the phylogeny (Figs. 1, 2, 3, 4 and 5). However, it requires coverage of at least 5-8X (depending on *k*-mer length) to ensure that not too many true *k*-mers are filtered out (Fig. 4). A pleasant side effect of filtering at the *k*-mer counting stage is that this decreases the size of *k*-mer table drastically and thereby decreases the computational load substantially (Table 1).

### Future directions

AAF could be used to develop automated phylogeny generators such as phylota [44] while using whole-genome data, or REALPHY [40] but not only for microbes. It can make use of both assemblies and sequencing reads that are available, as we demonstrated for primates (assemblies downloaded from ensemble and raw reads downloaded from the NCBI Short Reads Archive). Automation is made feasible by AAF’s robustness against incomplete and unequal coverage, and its ability to accept different read lengths from any sequencing platform. Although AAF is designed for phylogenetic analysis, it has wider application for understanding the pattern of relationships among different taxonomic levels of samples such as population structure within species. While AAF is mainly designed for large eukaryotic genomes, it is also able to analyze prokaryote datasets. Possible application of the alignment and assembly free approach requires further exploration for other types of sequencing data, such as RAD-seq and metagenomic data especially for organisms without a reference. Although AAF is designed for subjects without a good reference genome, studies on species with a reference will also benefit from AAFs computation efficiency and user-friendly pipeline. Because the AAF approach is based upon a matrix of genetic distances among the genomes, it is easy to add new data without recalculating all of the other distances among genomes.

Our overall AAF approach could also be expanded naturally to investigate different phylogenetic patterns within genomes. Our application of AAF to phylogenies generates “average” genetic distances between whole genomes and a corresponding phylogeny based on the overall differences among genomes. It is possible, however, to use AAF to identify suites of *k*-mers that are consistent with a different phylogeny from the majority. This compartmentalization of the evolutionary history of the genome could identify portions of the genome with discordant histories in comparison to the majority of the genome [25]. The approach could also be used, in conjunction with phenotypic information about species, to associate suites of *k*-mers with phenotypes of interest. An advantage of the overall AAF approach is that it can identify genomic elements (*k*-mers and contigs derived from them) that show interesting discordance or associative patterns among species without initially investing in whole-genome assembly and alignment. Thus, the use of AAF to construct phylogenies is only the initial step in a broader AAF program.

## Conclusions

The AAF method proved to be an accurate and efficient way of estimating the phylogenetic relationships using raw sequence data from whole genomes. We developed the theoretical basis for optimizing *k-*mer length selection, filtering, correcting tip branch lengths, and bootstrapping, directly addressing the problems of homoplasy, sequencing error, and incomplete coverage. Thus, AAF provides a robust tool for phylogeny reconstruction especially when only low-coverage and heterogeneous genome data are available – data that would challenge traditional assembly- and alignment-based methods.

## Methods

### Generating the *k*-mer table

We use programs from the *phylokmer* package [25] for counting and merging *k*-mers into a combined *k*-mer presence/absence table. The counting step identifies all possible *k*-mers for each genome. Adjacent *k*-mers overlap for (*k* – 1) consecutive nucleotides, so if there is complete coverage, each site is covered by *k k*-mers. When there is filtering, a *k*-mer is only recorded as present if it occurs as two or more copies in the same species. The merging step is to produce an *M* x *N* table of the counts of each of *M k*-mers among a group of *N* species. Because the number of *k*-mers counted for a given species will depend on the sequencing coverage and possible non-uniform coverage across the genome, the *k*-mer frequency table is converted to a table of presence/absence of *k*-mers among taxa.

### Estimating *d*

In our model, the evolutionary distance (*d*) is estimated from the proportion of *k*-mers that are shared between taxa. As in most evolutionary models, multiple substitutions at the same site limit the divergence among *k-*mers and hence the distance estimated between species [45]. Under the assumption that mutations only take the form of nucleotide substitutions, the probability that a given nucleotide undergoes *m* substitutions in distance *D* between two species is Poisson distributed; thus, this probability is *D*^*m*^*e*^*–D*^/*m*!. If γ_*m*_ denotes the probability that after *m* substitutions there is no change in the nucleotide, the probability of no change in the *k-*mer is

2 $$ \frac{n_s}{n_t}={\left({\displaystyle \sum_{m=0}^{\infty }{\gamma}_m\frac{D^m{e}^{-D}}{m!}}\right)}^k $$

Here, γ_0_ = 1, γ_1_ = 0, γ_2_ = *w*_*s*_^2^ + 2*w*_*t*_^2^, and γ_3_ = 2*w*_*s*_^2^*w*_*t*_ + 4*w*_*t*_^3^ where *w*_*s*_ and *w*_*t*_ are the probabilities of transitions and transversions. Values of γ_*m*_ for *m* > 3 are vanishingly small. Equation 2 can be solved to obtain the estimate *D*, although this estimate differs little from that given by Equation 1. For example, for *w*_*s*_ = 0.5 and *w*_*t*_ = 0.25, the difference between estimates *D* is 4 % when the true distance *d* = 0.2 and diminishes for lower values of *d* independently of *k* and genome size. Equation 2 assumes that all mutations are substitutions, and even though indels may be rare, large indels will lead to a much greater loss of shared *k-*mers than single substitutions. Therefore, multiple substitutions will in practice lead to even smaller differences in the estimate of *D* from Equation 1 in the main text. Thus, Equation 1 in the main text rather than Equation 2 above is included in the pipeline.

### Tree estimation

We constructed phylogenies from the *N* x *N* matrix of distance measures *D*_*ij*_ for species *i* and *j* using weighted least squares [1] with weights proportional to the expected variance of distances calculated for Equation 1. For fixed *n*_*t*_, the values of *n*_*s*_ will have an approximately binomial distribution with the number of trials *n*_*t*_ and the probability of success of each trial (persistence of a *k-*mer) given by *n*_*s*_/*n*_*t*_. Because *n*_*t*_ will be large, this can be approximated by a Gaussian distribution with mean *n*_*s*_ and variance *n*_*s*_(1 – *n*_*s*_/*n*_*t*_). From this, the distribution of *D*_*ij*_ will be approximately lognormal with variance proportional to

3 $$ \sigma \begin{array}{c}\hfill 2\hfill \\ {}\hfill D\hfill \end{array}\cong \frac{1}{n_t}\left(\frac{1-{e^{-k\widehat{D}}}^{{}_{ij}}}{e^{-k{\widehat{D}}_{ij}}}\right)=\frac{1}{n_t\omega \left(k{\widehat{D}}_{ij}\right)} $$

where ${\widehat{D}}_{ij}$ is the estimate of *D*_*ij*_ determined during the tree-fitting process. The phylogenetic tree for *N* species given by branch lengths ${\widehat{D}}_{ij}$ is that tree that minimizes ${\displaystyle \sum_{i,j}^N\omega}\left(k{\widehat{D}}_{ij}\right){\left({D}_{ij}-{\widehat{D}}_{ij}\right)}^2.$ For these calculations in our pipeline, we used the program fitch in the package PHYLIP [46], although we modified the program to accommodate the weights given by Equation 3.

### *k*-mer homoplasy

*K*-mer homoplasy is a central challenge for analyzing raw read data, and therefore we addressed it in detail. The results of the mathematical calculations of homoplasy are illustrated in Fig. 2. Here, we present the detailed mathematical results for interested readers.

To calculate the consequences of *k-*mer homoplasy on the estimated distance between species requires the distribution of *k-*mer frequencies within genomes. Let *Q*_*k*_ denote the random variable for the number of copies of a *k-*mer in a genome above 1; thus, if *q*_*k*_(*i*) denotes the probability distribution of *Q*_*k*_, *q*_*k*_(0) is the probability that a *k-*mer is unique, *q*_*k*_(1) is the probability that a *k-*mer occurs at two different locations in the genome, etc. There are three ways in which *k*-mer homoplasy increases the observed proportion of shared *k*-mers. (i) If there are multiple copies of a *k-*mer, then even if some copies undergo a mutation from species A to species B, the species will still share the *k-*mer if at least one copy in A does not undergo a mutation in B. (ii) Even if all copies of a *k*-mer mutate from species A to B, a mutation in another region of the genome of B could give rise to the same *k*-mer, so that this *k*-mer is still shared between A and B. (iii) Even if all of the copies of a *k-*mer in A undergo mutations, they will still be shared if the mutations generate *k-*mers that exist in B at different locations. Combining these three scenarios, the probability of two species sharing *k-*mers is *p*_*h*_ = *p*_1_ + (1 – *p*_1_)*p*_2_ + (1 – *p*_1_)(1 – *p*_2_)*p*_3_ where

4 $$ \begin{array}{l}{p}_1=1-{{\displaystyle \sum_i^{\infty}\left(1-{e}^{-kD}\right)}}^i\;{q}_k(i)\\ {}{p}_2=\left(1-{e}^{-kD}\right)\left[1-{\left(1-P\right)}^g\right]\\ {}{p}_3=0.5{e}^{-kD}{\displaystyle \sum_i^{\infty }{\left(1-{\left(1-P\right)}^g\right)}^i{q}_k(i)}\end{array} $$

Here, *P* is the probability of a given *k-*mer being identical at two different locations in the genome, which is 2(0.5 - *u* + *u*^2^)^*k*^ with GC content *u*, and *g* is the genome length. For theoretical exploration of the consequences of *k-*mer homoplasy, *p*_*h*_ can be calculated under the assumption that genomes are random sequences undergoing evolution. In this case, *Q*_*k*_ follows a binomial distribution with probability of success *P* and number of trials equal to the genome size, *g*. From these assumptions,

5 $$ \begin{array}{l}{p}_1=1-\frac{{\left(1-P{e}^{-kD}\right)}^g-{\left(1-P\right)}^g}{1-{\left(1-P\right)}^g}\\ {}{p}_3=0.5{e}^{-kD}\frac{{\left(1-P{\left(1-P\right)}^g\right)}^g-{\left(1-P\right)}^g}{1-{\left(1-P\right)}^g}\end{array} $$

The distribution *Q*_*k*_ gives the probability of homoplasy within a genome. *Q*_*k*_ can be calculated empirically from the *k*-mer frequency distribution. The two are the same for simulated (Fig. 2b, c) or assembled genome sequences (Fig. 2d). While calculating *Q*_*k*_ from sequencing reads, the *k*-mer frequency distribution needs to be corrected to account for coverage and sequencing errors (see below: Tutorial using 21 tropical trees).

### Tree comparison

We used both correlations between patristic distance matrices and the Branch Score Distance (BSD, from the PHYLIP package [46] to compare between our AAF optimal phylogeny (Fig. 1b) and the most recent phylogeny in the literature [31]. To compare phylogenetic trees produced from simulated SRS data (Table 2), we used BSD. BSD is based on the sum of the squared differences between the branch lengths of the two trees [32]. The larger the BSD, the larger is the distance between the trees. BSD has the advantage that it depends on absolute branch lengths rather than relative branch lengths (which determine correlations of patristic distances). Our mathematical results show that inaccuracies of the AAF method, such as the lengthening of branch tips (Eq. 10), often involve changes in absolute branch lengths. Therefore, BSD provides a rigorous test for comparisons between AAF trees.

### Missing *k*-mers due to incomplete coverage

Assuming that reads are random across a genome with coverage depth *c*, the probability of finding a *k-*mer with reads of length *r* is *p*_*r*_ = 1 – exp(−*L*) where *L* = *c*(*r* – *k* + 1)/*r*. If *k-*mers are filtered to remove singletons, then the probability of missing a *k-*mer due to coverage is *p*_*rf*_ = 1 – (1 + *L*)exp(−*L*). Note that the probability of recording a *k-*mer is lower for the case when singletons are filtered.

### Missing *k*-mers due to sequencing errors and filtering

We assume that sequencing errors replace a given nucleotide with A, T, C, or G at random with an error rate *E*. Then the probability of finding at least one copy of a true *k-*mer is

6 $$ {p}_e=1-\frac{ \exp \left(-L{\left(1-E\right)}^k\right)- \exp \left(-L\right)}{1- \exp \left(-L\right)} $$

If *k-*mers are filtered, the probability of including a true *k-*mer is

7 $$ {p}_{ef}=1\frac{\left(-L{\left(1-E\right)}^k\right)-\left(1+L\right) \exp \left(-L\right)}{1-\left(1+L\right) \exp \left(-L\right)}-{\left(1-E\right)}^k\frac{L \exp \left(-L{\left(1-E\right)}^k\right)}{1-\left(1+L\right) \exp \left(-L\right)}. $$

### False *k*-mers caused by sequencing errors

Sequencing errors will generate false *k-*mers that will increase the apparent number of *k-*mers within a given species, *n*_t_*. The probability that false *k-*mers are produced is approximately *p*_*ta*_ = *L*(1 – (1 – *E*)^*k*^). False *k-*mers will also increase the observed number of shared *k-*mers between species *n*_s_* by generating apparent homoplasy; the probability of generating a false shared *k-*mer is approximately *p*_*sa*_ = *L*(1 – (1 – *E*/3)^*kd*^). If *k-*mers are filtered, the probability of false *k-*mers generated by sequencing error is vanishingly small.

### Combined effects of coverage and sequencing error

The previous results make it possible to estimate the net effect of incomplete coverage and sequencing errors on the estimate of the distance between species. Let *p*_*t*_ and *p*_*s*_ denote the theoretically predicted ratios of observed to true total and shared *k-*mers, *n*_t_*/*n*_*t*_ and *n*_s_*/*n*_*s*_. In the absence of *k-*mer filtering, *p*_*t*_ = *p*_*r*_*p*_*e*_ + *p*_*ta*_ and *p*_*s*_ = *p*_*r*_^2^*p*_*e*_^2^ + *p*_*sa*_. In the presence of filtering, these equations also apply by replacing *p*_*r*_ and *p*_*e*_ by *p*_*rf*_ and *p*_*ef*_, and setting *p*_*ta*_ = *p*_*sa*_ = 0. These equations are used to show the effects of coverage, sequencing error, and filtering on the ratios *n*_t_*/*n*_*t*_ and *n*_s_*/*n*_*s*_ (Fig. 4). They can also be used to show the bias in estimates of distances caused by incomplete coverage and sequencing error (Fig. 5a). The distance computed from the observed total and shared *k-*mers is D* = −(1/*k*)log(*n*_s_*/*n*_t_*), and the difference between D* and *D* (that would be calculated if the true *n*_*t*_ and *n*_*s*_ were known) is

8 $$ D*-D=\frac{-1}{k} \log \frac{p_s}{p_t}. $$

To compute the loss of precision due to incomplete coverage and sequencing error, assume that the true numbers of shared and total *k-*mers, *n*_*s*_ and *n*_*t*_, are known; these values are random variables due to the evolutionary process, but we are interested only in the variation caused by incomplete coverage and sequencing error in estimating the distance between two species that represent a single realization of the evolutionary process. The variance in the estimate of *D* calculated from the observed ratio *n*_s_*/*n*_t_* is proportional to

9 $$ V\left[ \log \frac{n_{{}_S}^{*}}{n_t^{*}}\right]\cong \log \left(1+\frac{1}{n_s}\frac{q_s\left(1-{q}_s\right)+{p}_{sa}}{{\left({q}_s+{p}_{sa}\right)}^2}\right)+ \log \left(1+\frac{1}{n_s}\frac{q_t\left(1-{q}_t\right)+{p}_{ta}}{{\left({q}_t+{p}_{ta}\right)}^2}\right). $$

where *q*_*s*_ = *p*_*r*_^2^*p*_*e*_^2^ and *q*_*t*_ *= p*_*r*_*p*_*e*_ when singletons are not filtered, and *q*_*s*_ = *p*_*rf*_^2^*p*_*ef*_^2^, *q*_*t*_ *= p*_*rf*_*p*_*ef*_, and *p*_*ta*_ = *p*_*sa*_ = 0 when they are filtered. From this equation, precision always increases with coverage (Fig. 5b).

### Tip correction

Incomplete coverage and sequencing error will increase the estimated distance between species, but this bias can be corrected using Equation 8. Specifically, the tips of the tree can be reduced by

10 $$ {D}_{tip}=\frac{D*-D}{2}\cong \frac{1}{2k} \log \left(\frac{p_r{p}_e+{p}_{at}}{p_r^2{p}_e^2}\right). $$

if *k-*mers are not filtered; if *k-*mers are filtered, *p*_*r*_ and *p*_*e*_ are replaced by *p*_*rf*_ and *p*_*ef*_, and *p*_*ta*_ = 0. This approximation for *D*_*tip*_ depends only on *c*, *r*, *E* and *k*, and therefore if all species have similar values of *c*, *r* and *E*, the correction will be very similar for all species. Note that shortening tip lengths should be done only after the tree topology has been constructed, because the weights used in the least-squares tree estimation (Eq. 3) apply to the observed values *n*_t_* and *n*_s_*, not the corrected values.

When *c*, *r*, *E* and *k* differ greatly among species, the values given by Equation 10 will differ among tips. A solution to this situation is to calculate the average *D*_*tip*_ for all pairwise distances using the parameter values for taxa with the lower *n*_*t*_, and then trim each tip with the average of these values of *D*_*tip*_. This solution is preferable to correcting distances between taxa before constructing the phylogeny, because the construction of the phylogeny should incorporate variances due to coverage and sequencing error that would be removed by tip corrections.

### Bootstrapping

We developed a nonparametric bootstrap similar to standard bootstraps used for phylogenetic reconstructions, and also a parametric bootstrap that can be scaled to very large genomes. Both bootstraps separate the effects of sampling variation (incomplete coverage, sequencing error) and evolutionary variation (mutations).

The nonparametric bootstrap first randomly resamples reads (with replacement) to assess the uncertainty in the phylogenetic tree caused by sampling variability. For each resampled data set, a *k-*mer table is generated. To isolate the effect of sampling variability alone, bootstrap phylogenies are constructed from these *k-*mer tables. To include evolutionary variability, the second stage takes each *k-*mer table generated from resampling reads and resamples the table (with replacement) by taking rows with probability 1/*k*; this follows the “block bootstrap” proposed by [47, 48]. This resampling shortens the *k-*mer table by 1/*k* to account for the overlap between *k-*mers; each nucleotide can be covered by *k k-*mers. In simulations of sequence evolution, this resampling procedure gave good approximations to the evolutionary variance in *D* estimated between two species, validating the block bootstrap for this application.

The parametric bootstrap uses our equations for the variation in the estimates of distances to simulate genetic distances including sampling and evolutionary variation. For stage one, the approach involves adding a random variable e_*ij*_ to the distance between each pair of species *i* and *j*, thereby “contaminating” the distance matrix with the uncertainty expected from incomplete coverage and sequencing error; e_*ij*_ is selected from a normal random number generator with mean zero and variance

$$ \log \left(1+\frac{1}{n_s}\frac{q_s\left(1-{q}_s\right)+{p}_{sa}}{{\left({q}_s+{p}_{sa}\right)}^2}\right)+ \log \left(1+\frac{1}{n_s}\frac{q_t\left(1-{q}_t\right)\left(1+\left(r-k\right)/L\right)+{p}_{ta}\left(1+w\right)}{{\left({q}_t+{p}_{ta}\right)}^2}\right) $$

11 $$ \mathrm{where}\ \mathrm{w}={\displaystyle \sum_{i=1}^{k-1}\frac{2i}{k\left(r-k+1\right)} \max \left(0,r-2k+i+1\right)} $$

Simulations showed that this formula sometimes under-estimates and sometimes over-estimates the true standard deviation in the distance between taxa by as much as 50 %. To provide a conservative bootstrap (i.e., one that is not going to improperly inflate the support for nodes), we multiplied the estimated standard deviation of e_*ij*_ by a correction factor of 2. The resulting distance matrix is then used to construct a bootstrap phylogenetic tree. Repeating this procedure 100 (or more) times gives an estimate of the proportion of branch nodes that are consistent despite uncertainty in genetic distances between species caused by incomplete coverage and sequencing error. For stage two, the “contaminated” distance matrix from stage one is contaminated a second time to account for variation in mutations by adding the random variable e_*ij*_ with mean zero and variance given by

12 $$ \frac{1}{k^2{n}_t}\left[\left({e}^{kD}-1\right)+2{\displaystyle \sum_{i=1}^{k-1}\left({e}^{iD}-1\right)}\right] $$

This equation is modified from Equation 3 to incorporate covariances caused by overlapping *k*-mers. The second stage of parametric bootstrap also incorporates covariances among distances between species that share a common branch in the tree. These covariances are estimated from the phylogenetic tree computed from the original data so that the covariance between distances d(A,B) and d(C,D) equals the proportion of evolutionary history shared by the four species (i.e., the distance between the node separating A and B, and the node separating C and D).

### Simulation of sequence evolution

We used Rose (Random-model Of Sequence Evolution [49]) to simulate sequences under a HKY model of evolution [50] with a transition bias of 2. We assumed that the insertion and deletion rates were the same, and that their sum was 10 % of the substitution rate. Both insertions and deletions had length uniformly distributed between 1 and 5. There are two types of simulations in our study: simulations of differences in sequences between two species given a true distance *d* between them, and simulations of many sequences from a phylogeny. The first is used to assess the estimate of distance, *D* (Fig. 3a, b), and the second is used to assess the ability of AAF (Fig. 3c) and *co-phylog* (Additional file 7: Figure S3) to recover the phylogeny used as starting trees. We used the phylogeny given in Fig. 1b as the starting tree and randomly selected sequences from a segment of the rabbit genome [30] as the ancestral genome sequences.

### Read simulations

The primate genome assemblies were downloaded from the Ensembl database [50], and we simulated pair-end Illumina data using Dwgsim (Whole Genome Simulation, http://sourceforge.net/apps/mediawiki/dnaa/) assuming a read length of 70 bp, a sequencing error rate of 1 %, and coverages of 2X and 5X, with and without filtering.

### Availability of supporting data

The SRS data sets of the 21 tropical trees are available in the NCBI Short Reads Archive. See accession numbers in Additional file 5: Table S3.

## Additional files

Additional file 1: Figure S1. Primate phylogeny reconstructed by AAF from assembled genomes or simulated reads. From genome assemblies with (A) k = 15 (B) k = 23 (C) k = 25 (D) k =31. (E) Using 70-bp reads simulated from assembled genomes with 1 % sequencing errors and 0.5 coverage. Incorrect branches (relative to the optimal tree with k = 21) are shown in red. Additional file 2: Table S1. Branch Score Distance (BSD) between the optimal AAF tree and trees generated with larger k. Additional file 3: Table S2. General information and accession numbers in NCBI Short Reads Archive of the mammal dataset. Additional file 4: Figure S2. Phylogeny of mammals constructed with raw reads downloaded from NCBI Short Reads Archive. (A) 7 primates. (B) 12 mammals. See details of dataset in Additional file 3: Table S2. Additional file 5: Table S3. General information and accession numbers of the 21 tropical trees dataset. Additional file 6:
**Tutorial of the analysis of the 21 tropical trees dataset.** Contains the whole process from obtaining data to parameter selection, phylogeny reconstruction and bootstrap. Additional file 7: Figure S3. Performance comparison between AAF and *co-phylog* measured by the number of topological mistakes in the phylogeny for initial sequence lengths ranging from 5 to 640 KB. Rose 1.3 was used to simulate sequence evolution down the 12 species phylogeny given in Fig. 1b (see Methods: Simulation of sequence evolution); for each sequence length, 100 simulations were performed, and the number of topological mistakes averaged. For Co-phylog, the values of *CK* (length of the flanking regions) were 4 (red), 5 (orange), 6 (purple), 7 (green) and 8 (cyan), and for AAF the values of *k* were 11 (blue dots) and 13 (black dots). Additional file 8: Figure S4. Comparison of pairwise distances for the tropical trees dataset calculated with different *k*. Additional file 9: Figure S5. Estimating the optimal *k* of the tropical trees dataset. Theoretical predictions of the proportion of shared *k*-mers, *ph*, calculated from the observed frequency distribution of *k*-mers, *Q*_*k*_, for the tropical trees dataset ranging in size from 400 M to 1.3Gbp assuming the true distance between taxa is d = 0.1 (divergence time 94Mya).

## Abbreviations

AAF: Assembly and Alignment-Free
BSD: Branch Score Distance
SRS: Short Read Sequences
