# Supplementary figures and images for: An assembly and alignment-free method of phylogeny reconstruction from next-generation sequencing data
Source: BMC Genomics. 2015 Jul 14;16(1):522. doi: 10.1186/s12864-015-1647-5 (PMC4501066; doi:10.1186/s12864-015-1647-5)

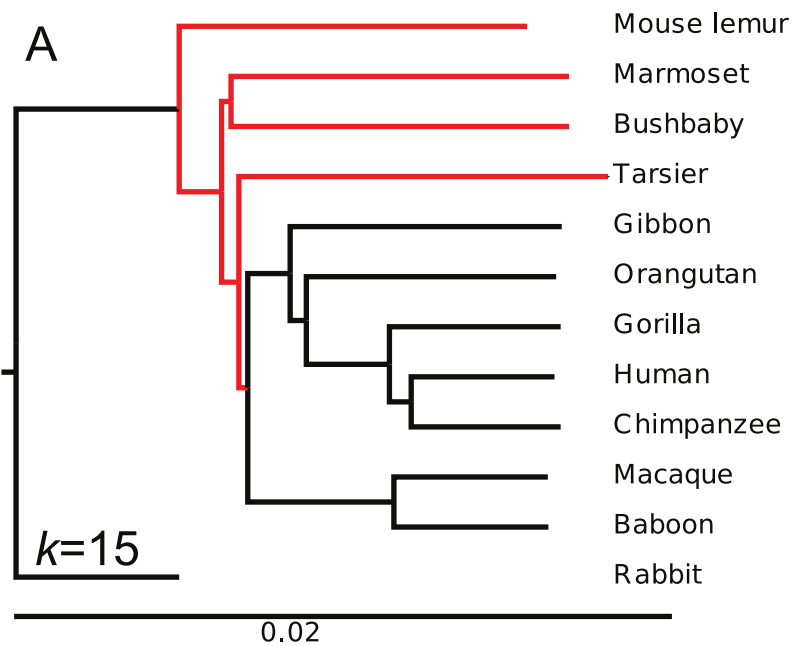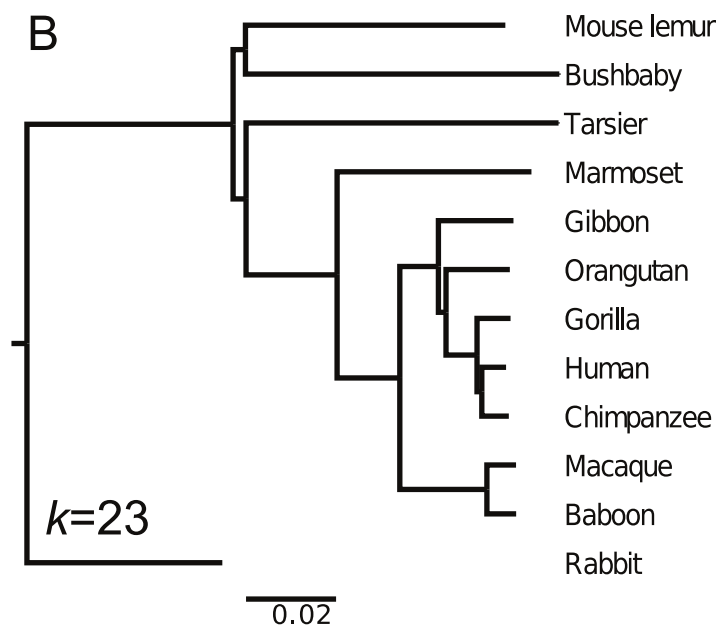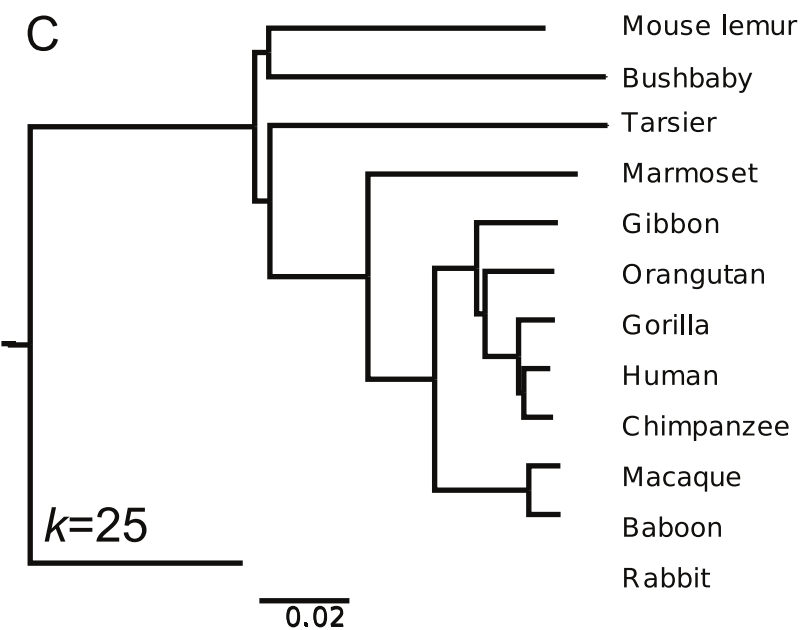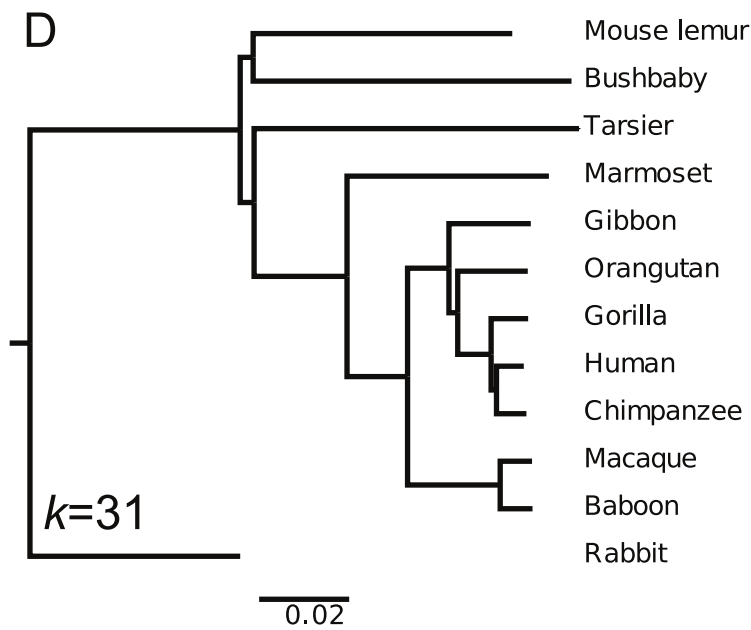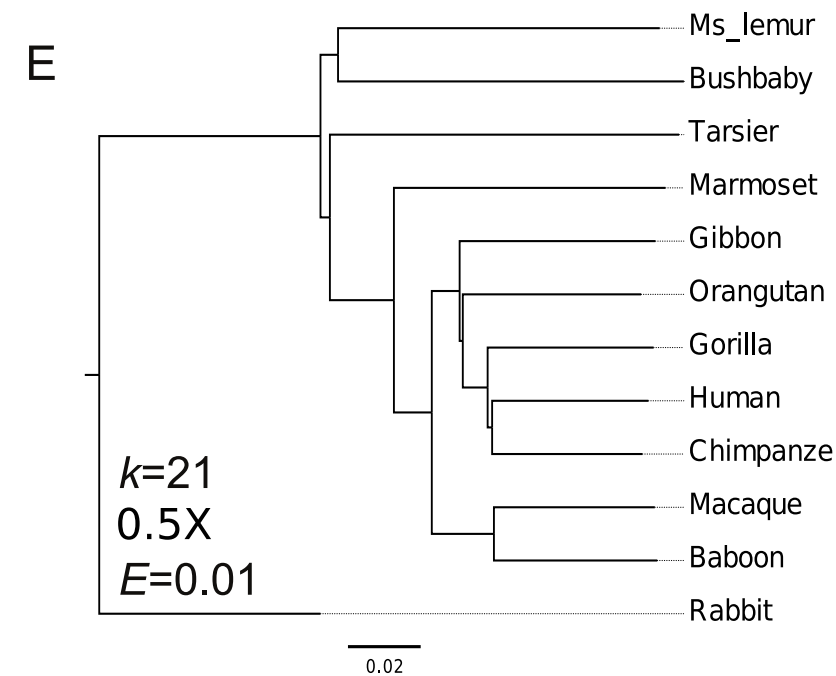

— Incorrect branches

Supplement: Additional file 1: Figure S1. — Primate phylogeny reconstructed by AAF from assembled genomes or simulated reads. From genome assemblies with (A) k = 15 (B) k = 23 (C) k = 25 (D) k =31. (E) Using 70-bp reads simulated from assembled genomes with 1 % sequencing errors and 0.5 coverage. Incorrect branches (relative to the optimal tree with k = 21) are shown in red. [file 12864_2015_1647_MOESM1_ESM.pdf]

A

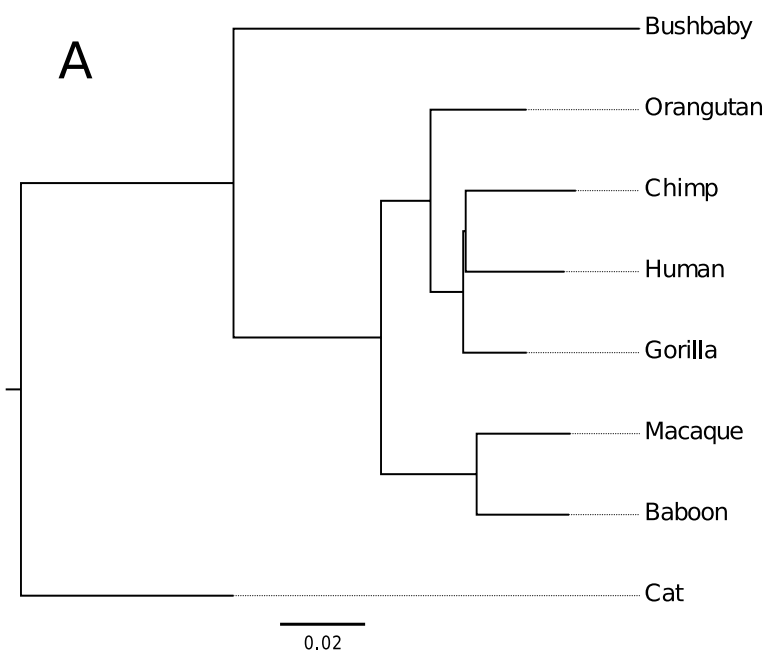

B

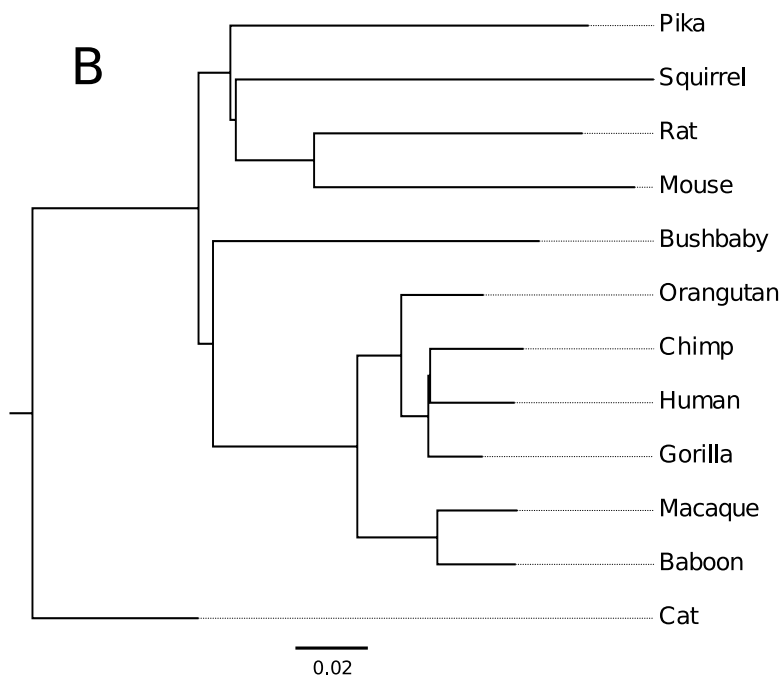

C

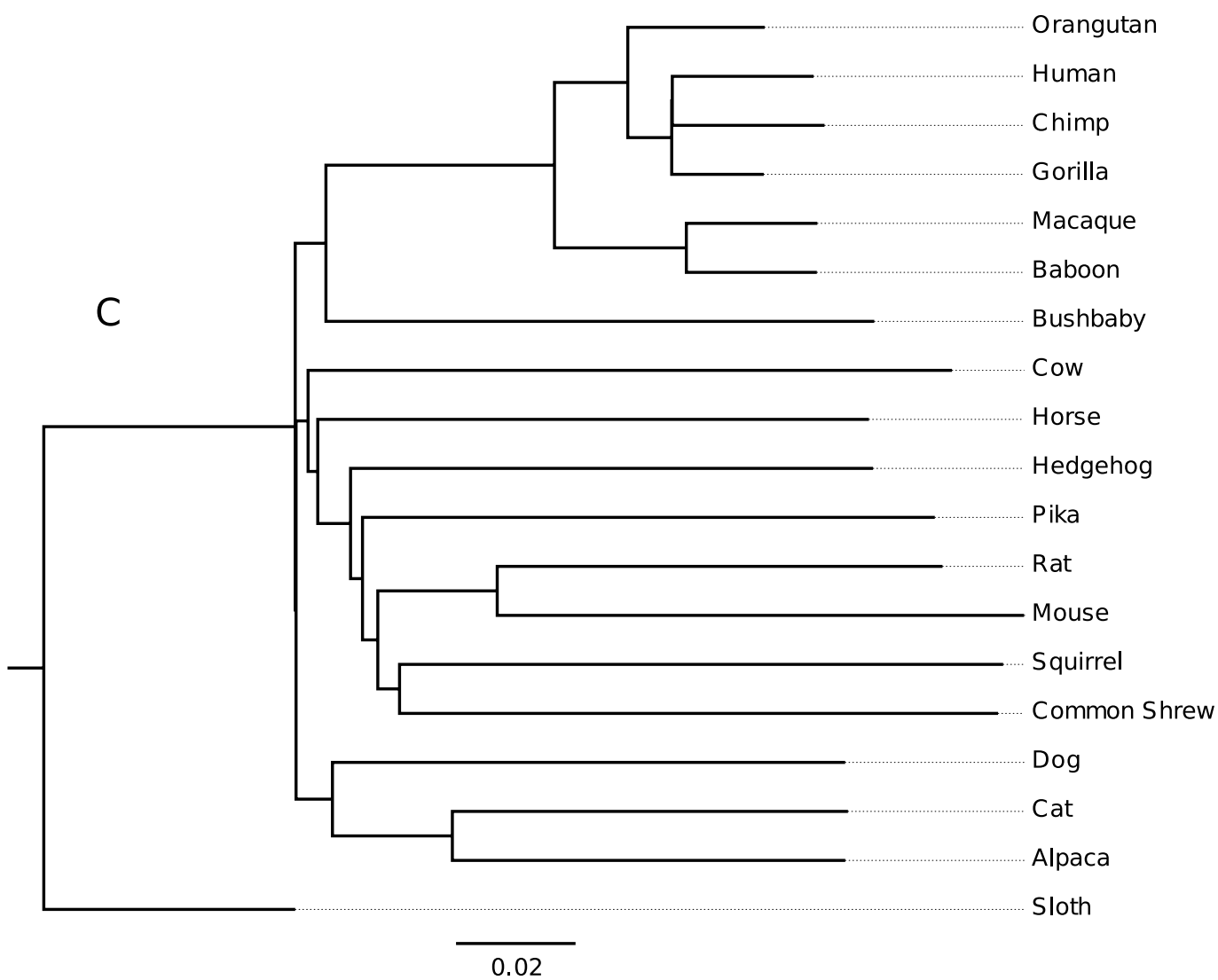

Supplement: Additional file 4: Figure S2. — Phylogeny of mammals constructed with raw reads downloaded from NCBI Short Reads Archive. (A) 7 primates. (B) 12 mammals. See details of dataset in Additional file 3: Table S2. [file 12864_2015_1647_MOESM4_ESM.pdf]

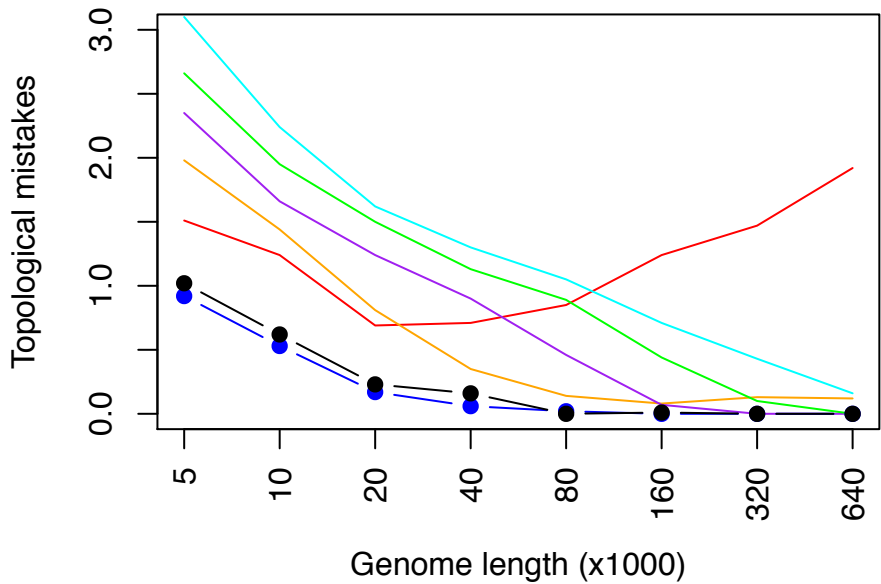

Supplement: Additional file 7: Figure S3. — Performance comparison between AAF and co-phylog measured by the number of topological mistakes in the phylogeny for initial sequence lengths ranging from 5 to 640 KB. Rose 1.3 was used to simulate sequence evolution down the 12 species phylogeny given in Fig. 1b (see Methods: Simulation of sequence evolution); for each sequence length, 100 simulations were performed, and the number of topological mistakes averaged. For Co-phylog, the values of CK (length of the flanking regions) were 4 (red), 5 (orange), 6 (purple), 7 (green) and 8 (cyan), and for AAF the values of k were 11 (blue dots) and 13 (black dots). [file 12864_2015_1647_MOESM7_ESM.pdf]

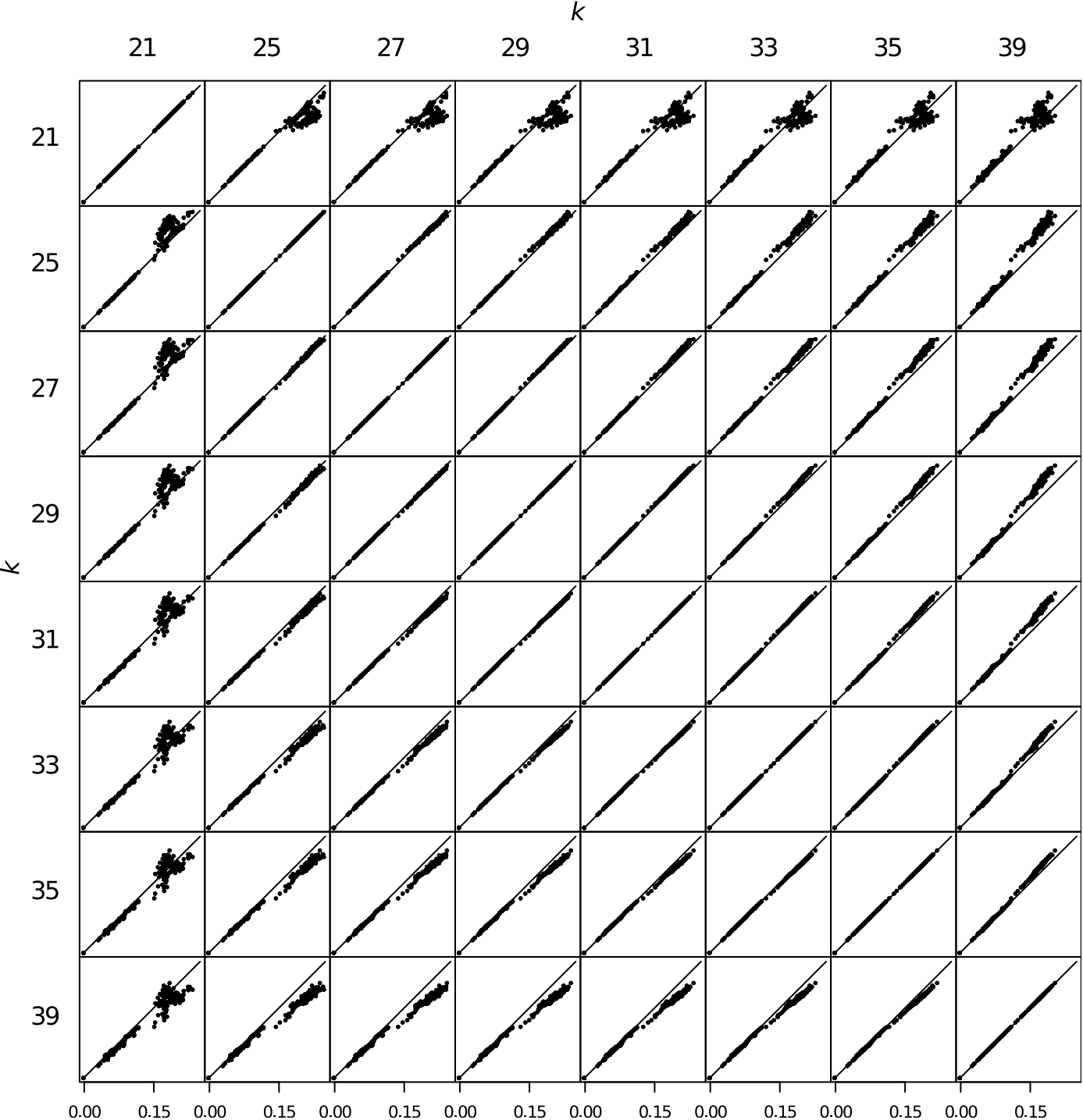

Supplement: Additional file 8: Figure S4. — Comparison of pairwise distances for the tropical trees dataset calculated with different k. [file 12864_2015_1647_MOESM8_ESM.pdf]

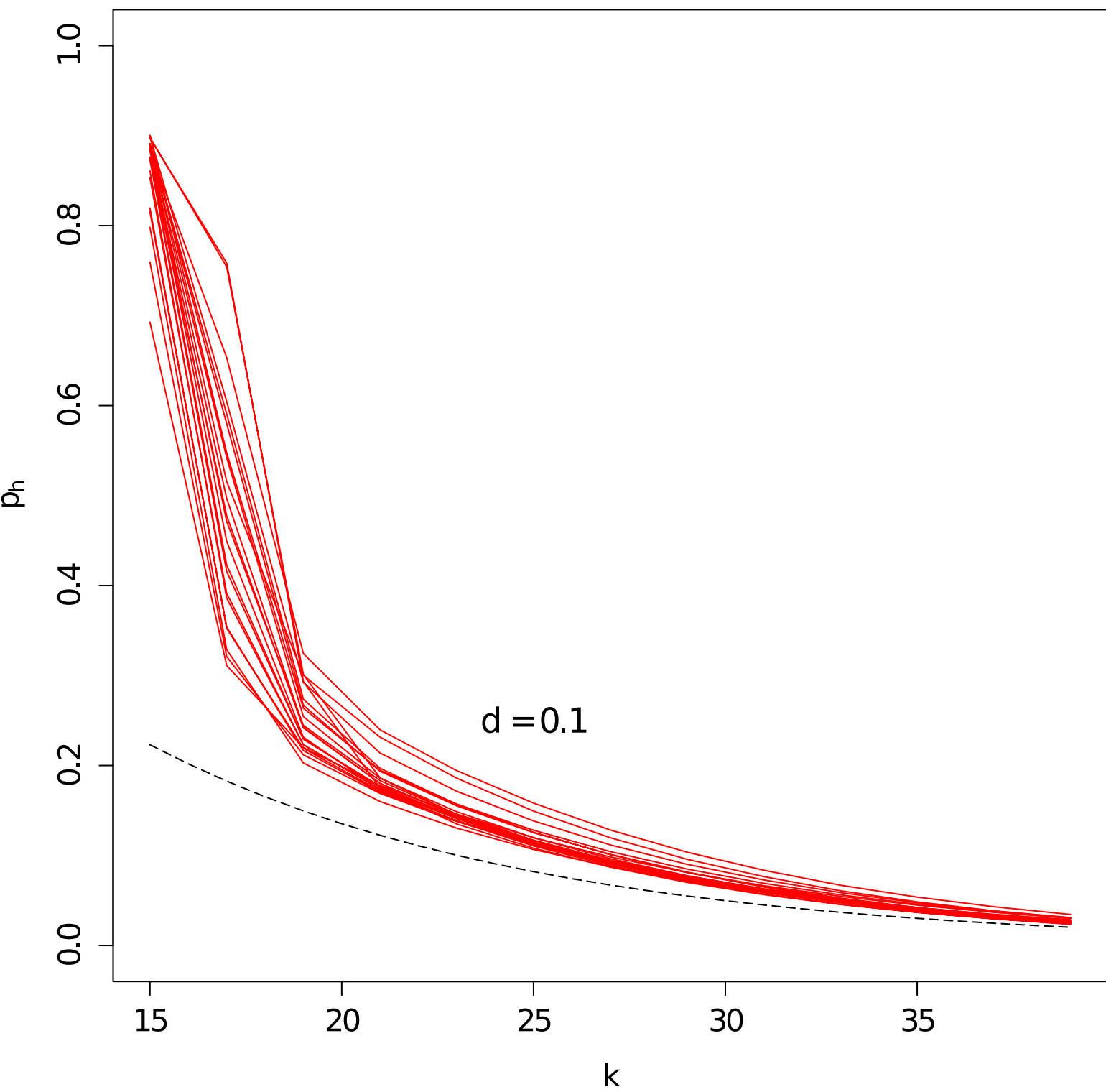

Supplement: Additional file 9: Figure S5. — Estimating the optimal k of the tropical trees dataset. Theoretical predictions of the proportion of shared k-mers, ph, calculated from the observed frequency distribution of k-mers, Q k, for the tropical trees dataset ranging in size from 400 M to 1.3Gbp assuming the true distance between taxa is d = 0.1 (divergence time 94Mya). [file 12864_2015_1647_MOESM9_ESM.pdf]
