# Supplementary material for: An assembly and alignment-free method of phylogeny reconstruction from next-generation sequencing data
Source: BMC Genomics. 2015 Jul 14;16(1):522. doi: 10.1186/s12864-015-1647-5 (PMC4501066; doi:10.1186/s12864-015-1647-5)
Supplement: Additional file 2: Table S1. — Branch Score Distance (BSD) between the optimal AAF tree and trees generated with larger k. [file 12864_2015_1647_MOESM2_ESM.pdf]

Table S1: Branch Score Distance (BSD) between the optimal AAF tree ($k = 21$) generated from assembled data of the 11 primate dataset (Fig. 2B) and trees generated with larger k.

Tree	BSD
$k = 23$	0.014
$k = 25$	0.014
$k = 27$	0.011
$k = 31$	0.007
