# Supplementary material for: An assembly and alignment-free method of phylogeny reconstruction from next-generation sequencing data
Source: BMC Genomics. 2015 Jul 14;16(1):522. doi: 10.1186/s12864-015-1647-5 (PMC4501066; doi:10.1186/s12864-015-1647-5)
Supplement: Additional file 3: Table S2. — General information and accession numbers in NCBI Short Reads Archive of the mammal dataset. [file 12864_2015_1647_MOESM3_ESM.pdf]

Table S2: General information and accession numbers in NCBI Short Reads Archive of the mammal dataset

Common name	Latin name	Read length (bp)	Total bp(MB)	Coverage	Sequencing platform	Accession
Orangutan	*Pongo abelii*	100	18,965	5.39	Illumina HiSeq 2000	SRR748032
Gorilla	*Gorilla gorilla*	100	9,404	2.73	Illumina HiSeq 2000	SRR748194
Human	*Homo sapiens*	100	6,969	2.04	Illumina HiSeq 2000	ERR251906
Chimpanzee	*Pan troglodytes*	101	7,056	2.09	Illumina Genome Analyzer II	ERR032961
Macaque	*Macaca mulatta*	49	9,513	2.76	Illumina Genome Analyzer II	SRR278740
Baboon	*Papio anubis*	95	6,383	1.85	Illumina Genome Analyzer IIx	SRR927656
Bushbaby	*Otolemur garnettii*	101	3,216	0.91	Illumina Genome Analyzer II	SRR016879
Cat	*Felis catus*	563	5,091	1.79	454 GS FLX Titanium	SRR069335- SRR069379
Pika	*Ochotona princeps*	110	9,800	2.05	Illumina HiSeq 2000	SRR402203- SRR402205 SRR402207
Squirrel	*Spermophilus tridecemlineatus*	101	18,000	7.26	Illumina HiSeq 2000	SRR305235 SRR305236 SRR317823
Rat	*Rattus norvegicus*	91	10,200	3.51	Illumina Genome Analyzer II	SRR351210
Mouse	*Mus musculus*	37	13,900	5.01	Illumina HiSeq 2000	SRR091261
Horse	*Equus przewalski*	99	13,269	5.36	Illumina HiSeq 2000	SRR896620 SRR896621 SRR896641 SRR896644 SRR899939
Hedgehog	*Erinaceus europaeus*	102	10,000	2.96	Illumina HiSeq 2000	SRR396597
Alpaca	*Vicugna pacos*	501	7,561	2.55	454 GS FLX Titanium	SRR531044- SRR531070
Common Shrew	*Sorex araneus*	102	11,100	3.77	Illumina HiSeq 2000	SRR278622
Dog	*Canis lupus familiaris*	45	5,500	2.28	Illumina Genome Analyzer IIx	SRR782085
Sloth	*Choloepus hoffmanni*	101	20,500	8.31	Illumina HiSeq 2000	SRR866928
