# Supplementary material for: An assembly and alignment-free method of phylogeny reconstruction from next-generation sequencing data
Source: BMC Genomics. 2015 Jul 14;16(1):522. doi: 10.1186/s12864-015-1647-5 (PMC4501066; doi:10.1186/s12864-015-1647-5)
Supplement: Additional file 5: Table S3. — General information and accession numbers of the 21 tropical trees dataset. [file 12864_2015_1647_MOESM5_ESM.pdf]

Table S3: General information and accession numbers of the 21 tropical trees dataset.

Species	Code	Read Length (bp)	Total Base Pair	Coverage	Accession
Intsia bijuga	IB	76	9,170,193,440	5.49	SRX596332
Intsia palembanica (1 individual)	IP1	76	10,185,026,760	5.99	SRX600007
Intsia palembanica (10 individual pooled)	IP2	76	9,910,279,616	4.79	SRX600313
Castanopsis echinocarpa	CE	51	2,055,720,648	1.84	SRX017482
Castanopsis indica	CI	51	1,992,413,430	1.80	SRX017483
Castanopsis sp.	CS	51	2,051,372,694	4.03	SRX602174
Lithocarpus balansae	LB	51	1,759,605,978	1.62	SRX016680
Lithocarpus calolepis	LC	51	2,095,070,004	1.64	SRX017436
Lithocarpus fenestratus	LF	76	9,774,729,816	9.22	SRX601832
Lithocarpus grandifolius	LG	51	2,001,160,644	1.71	SRX017338
Lithocarpus hancei	LH	51	3,243,525,846	2.79	SRX017339
Lithocarpus craibianus	LR	76	8,993,204,336	6.39	SRX601833
Lithocarpus xylocarpus	LX	51	2,693,888,340	2.39	SRX017340
Trigonobalanus doichangensis	TD	51	3,574,391,304	3.92	SRX017683
Ficus altissima	FA	51	2,230,794,570	4.75	SRX017643
Ficus fistulosa	FF	51	894,202,584	3.58	SRX016302
Ficus langkokensis	FL	51	2,054,006,640	5.81	SRX017645
Ficus microcarpa	FM	51	1,487,138,886	4.24	SRX017740
Ficus racemosa	FR	76	9,068,985,456	8.99	SRX601830
Ficus tinctoria	FT	51	4,828,850,952	7.02	SRX017840
Ficus vasculosa	FV	76	9,638,585,392	11.68	SRX600314
Gonystylus bancanus	GB	51	2,265,978,756	4.41	SRX012692-94
