# Supplementary material for: An assembly and alignment-free method of phylogeny reconstruction from next-generation sequencing data
Source: BMC Genomics. 2015 Jul 14;16(1):522. doi: 10.1186/s12864-015-1647-5 (PMC4501066; doi:10.1186/s12864-015-1647-5)
Supplement: Additional file 6: — Tutorial of the analysis of the 21 tropical trees dataset. Contains the whole process from obtaining data to parameter selection, phylogeny reconstruction and bootstrap. [file 12864_2015_1647_MOESM6_ESM.pdf]

**Tutorial using 21 tropical trees**

This tutorial demonstrates the application of the AAF (Alignment and Assembly Free) package on a dataset consisting of Illumina sequences from 21 tropical trees. Using the tutorial we hope to give a detailed description of how AAF works with real-world problems.

**1) Data preparation**

1a. Download the read data from NCBI Short Reads Archive (SRA) (http://www.ncbi.nlm.nih.gov/sra) according to the accession number of each species given in Table S3.

1b. Convert the SRA files into fastq/fasta format.

A copy of fastq-dump from the SRA toolkit is included in the tropical_trees folder:

```
$ ./fastq-dump --split-3 *.sra
```

The whole dataset is about 300GB. If you are worried about your disk space, you can convert the fastq files into fasta files (given that the base quality is not considered in AAF) and compress them into .gz files. Both kmer_count and filter are capable of processing .gz files.

To convert files to fasta and compress them:

```
$ ./fastq-dump --fasta --split-3 --gzip *.sra
```

1c. Prepare the data structure for phylokmer.

Sequence files for each taxa need to be in one directory named after its respective taxon/sample. For example, there are two runs for *Ficus altissima*, SRR037748 and SRR037749. We create a folder for *Ficus altissima*, named "FA" for short, and move the data for the two runs inside. Therefore, there will be *N* directories for *N* samples, and the name of the directories will be the names displayed in the final phylogenetic tree. Put all the sample folders into a new directory, which will be your data directory requested by AAF_phylokmer.pl. Note that we have already created all the folders in the right structure (path_to_AAF/AAF/tropical_trees/data). The users only need to put the data downloaded from NCBI into the right species folder.

Within the tutorial package we have already prepared the data directory called “data”. You just need to move the sequences you downloaded for each sample into their folders. Note that there are two samples for *Intsia palembanica*, IP0001 (IP1) and IP0002 (IP2).

**2) Parameter selection**

2a. Selecting the optimal $k$

The optimal $k$ is selected using $p_h$ given in Figure S6, which requires the following information:

i. Coverage

The coverage for the tropical trees is calculated in two ways. For species with published genome size (CE, CI, LB, LC, LF, LG, LH, LX, TD. Chen (in press) *Tree Genetics and Genomes*), we calculated the coverage by dividing the total number of base pairs by the genome size. For the rest we estimated the $k$-mer coverage from the total number of $k$-mers (including multiple copies of the same $k$-mer) divided by $k$-mer diversity (number of $k$-mer that shows up at least once). Then we calculated the base coverage from $k$-mer coverage according to the equation

base_coverage = *k*-mer_coverage * read_length / (read_length – *k*-mer_size + 1)
Note that there are other, more accurate methods for estimating coverage. However, the selection of the optimal *k* does not depend strongly on the estimate of coverage, so this approximate approach is adequate.

ii. Genome size
The genome size is either known or calculated by dividing the total number of base pairs by the coverage estimated from the previous step.

iii. GC content
We calculated the GC content for each sample using gc.py in the utils folder. For example for CE:
$python gc.py AAF/tropical_trees/data/CE fastq

iv. Genetic distance (*d*)
We used 0.1, the default *d* for ploting Figure S6. This also matches the average distance in the distance matrix of *k*27. In practice, the value of *d* mainly serves to scale the value of the estimates of $p_h$ (i.e., scales the vertical axis of Fig. S6) and therefore does not have a large effect on the selection of the optimal *k*.

v. Frequency distribution of *k*-mers in the genome ($Q_k$)
We used jellyfish to calculate the *k*-mer frequency distribution for $k \leq 31$, and for $k>31$ we calculated the $Q_k$ from the pkdat files using pkdat2hist.py in the utils folder. For assembled data, the *k*-mer frequency table is the same as $Q_k$. For unassembled data that have multiple *k*-mers covering the same location on the genome, it is necessary to approximate $Q_k$. We did this by removing all singleton *k*-mers, as these will likely arise from sequencing error, and then binned the frequency distribution of *k*-mers according to the coverage. For example, for a genome with 5X coverage, *k*-mers were binned into categories 2-5, 6-10, 11-15, etc. These bins were then used for the frequency distribution $Q_k$. Although this approach is clearly only a rough approximation of the true frequency distribution $Q_k$, the selection of the optimal *k* does not depend sensitively on $Q_k$.

With the information from i-v above, we constructed the $p_h$ vs. *k* figure (Fig. S6) using the R code phVSk.R in the utils folder. The trend for all the red lines (estimated ph based on the $Q_k$ for each species) stabilized for $k \geq 25$, and the difference between the red lines and the black dashed line continued to decrease with larger *k*. Therefore, we constructed phylogenies for *k* from 25 to 31, and because the phylogenies were identical for $k \geq 27$ (Fig. 7), we selected 27 as the optimal *k* for the tropical trees dataset. The same phylogenetic topology was also obtained when *k*-mers were filtered to remove singletons. For *k* greater than 31, the topology within the *Ficus* group showed some small changes. We suspect that this is due to the loss of sensitivity to evolutionary changes when selecting *k*-mer lengths too long, especially for relatively small genomes (as the *Ficus* group has genome sizes less than half those of the other species).

2b. Filter or not

We chose not to filter since more than half of the species have coverage <5X. However, when we did filter, we obtained the same phylogenetic topology.

**3) Run (see details of output files in the AAF documentation)**

Counting *k*-mers:

path_to_AAF/AAF$ nohup python aaf_phylokmer.py -k 27 -d tropical_trees/data -G 16 -f FQ > aaf_phylokmer.log 2>&1 &

Constructing the phylogenetic tree:

path_to_AAF/AAF/$ nohup python aaf_distance.py -i tropical_trees/data/phylokmer.dat.gz -o tropical_trees -t 4 -m 16 -f tropical_trees/data/kmer_diversity.wc > aaf_distance.log &

**4) Tip correction (optional)**

The additional information that is needed for tip trimming beyond those needed to select the optimal *k* is the sequencing error rate. Illumina claims that the error rate for Genome Analyzer is about 1% (http://res.illumina.com/documents/products/datasheets/datasheet_genomic_sequence.pdf). We have prepared tip_info_tt.txt as the infomation file. To run the tip trimming, use the command: path_to_AAF/AAF/$ python aaf_tip.py -i tropical_trees/tropical_trees.tre -k 27 --tip tropical_trees/tip_info_tt.txt -f tropical_trees/data/kmer_diversity.wc

**5) Bootstrap**

The tropical tree dataset is too large for a nonparametric bootstrap. Therefore, we performed a parametric bootstrap using parametric_bootstrap_tt.R. The supporting rate for each node was 100%.

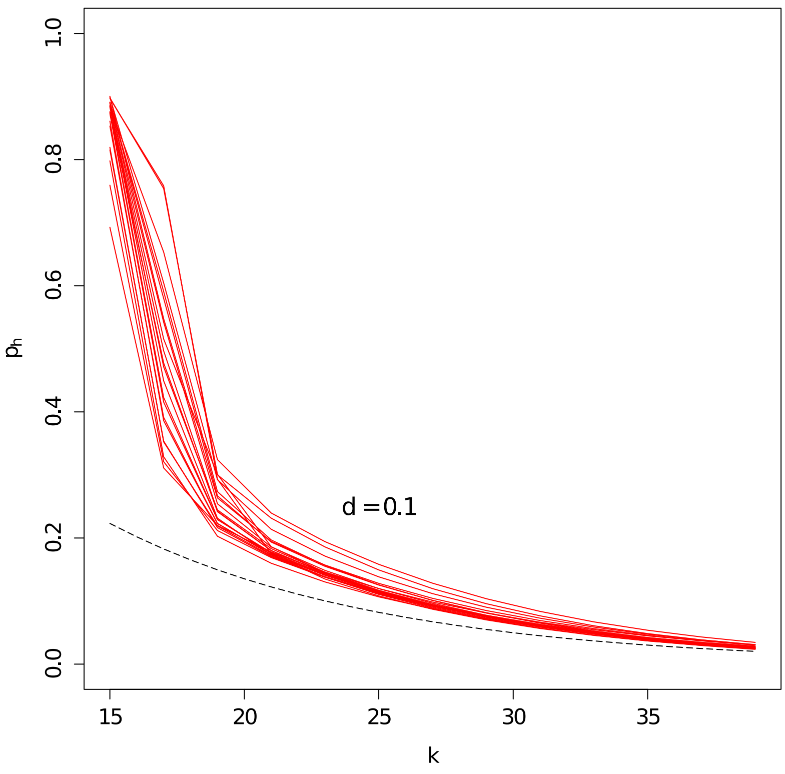


**Figure S5. Estimating the optimal k of the tropical trees dataset.** Theoretical predictions of the proportion of shared k-mers, ph, calculated from the observed frequency distribution of k-mers, Qk, for the tropical trees dataset ranging in size from 400M to 1.3Gbp assuming the true distance between taxa is d = 0.1 (divergence time 94Mya).
